# Supplementary material for: Fully intravenous split-dose administration of the oncolytic adenovirus TILT-123 in advanced solid tumors
Source: Mol Ther. 2026 May 13;34(8):4670–86. doi: 10.1016/j.ymthe.2026.05.007 (PMC13464138; doi:10.1016/j.ymthe.2026.05.007)
Supplement: Supplementary file 1 [file mmc1.pdf]

YMTHE, Volume *34*

Supplemental Information

Fully intravenous split-dose administration of the oncolytic adenovirus TILT-123 in advanced solid tumors

Elise Jirovec, Katriina J. Jalkanen, Dafne C.A. Quixabeira, James H.A. Clubb, Tuomo Alanko, Tatiana V. Kudling, Victor Arias, Santeri A. Pakola, Susanna Grönberg-Vähä-Koskela, Mirte van der Heijden, Anniina Färkkilä, Juha Kononen, Jorma Sormunen, Jukka Kemppainen, Maria Hellesuo, Lyna Haybout, Claudia Kistler, Suvi Sorsa, Riikka Havunen, Joao M. Santos, Anna Kanerva, Victor Cervera-Carrascon, Otto Hemminki, and Akseli Hemminki

Table S1. PET-based criteria used for evaluation of antitumor efficacy.

Complete metabolic response (CMR)	Complete resolution of fluorodeoxyglucose (FDG) activity within measurable lesions and all reliably assessable lesions to background levels. No new FGD-avid lesions in pattern typical of cancer*. Lymph nodes may remain metabolically active due to immune response (activated lymphocytes take up FDG)
Partial metabolic response (PMR)	>30% reduction in FDG activity measured as the summed SUVmax of measurable lesions (up to five lesions, max 2/organ). No new FGD avid lesions in pattern typical of cancer*.
Minor metabolic response (MMR)	10-29% decrease in FGD summed SUVmax. No new FDG-avid lesions in pattern typical of cancer*.
Stable metabolic disease (SMD)	0-9% decrease or up to <30% increase in FDG summed SUVmax. No new FDG-avid lesions in patten typical of cancer*.
Progressive metabolic disease (PMD)	≥30% increase in FDG summed SUVmax in pattern typical of tumor, or new clearly FDG-avid clinically significant lesions in pattern typical of cancer*.

* Defined as lesions associated with CT abnormality most consistent with cancer ≥2 cm in diameter, and clearly not because of inflammation related to treatment response.

Table S2. Summary of collected and analyzed tumor biopsies. Each column reflects individual biopsy results per day of collection for a specific assay. The final column summarizes overall positivity, defined as a positive result in at least one of the three assays. + indicates a collected biopsy or positive result, - indicates not collected or negative result.

Patient ID	Tumor biopsy location	Tumor biopsy collected D-14/D8/D43	TILT-123 DNA detection by qPCR	Hexon detection by IHC	Immune cell infiltration by mIF	Positivity in any assay
20114	Thorax	+/+/-	Excluded - no tumor present			
20115	Liver	+/+/-	D8+	D8-	D8+	D8+
20116	Lung	+/+/+	D8- D43 -	D8- D43-	D8+ D43+	D8+D43+
20220	Liver	+/+/+	D8+ D43-	D8+ D43+	D8- D43+	D8+D43+
20221	Liver	+/*/+	D22- D43-	D22- D43-	D22+ D43-	D22+D43-
20222	Liver	+/+/-	D8-	D8-	D8+	D8+

*Biopsy taken on D22 instead of D8

Table S3. Primer and probe sequences used for qPCR detection of TILT-123 DNA.

	Target Sequence
Forward primer	5'- CATGCTTTACA TGTGTTTAGTC GAG-3'
Reverse primer	5'- TAGTGCAATGC AAGACAGGAG-3'
Probe	5'-/56- FAM/TTGCATCC T/ZEN/GTACATG GTTGTGGC/3IABkFQ/ -3'

Table S4. Antibodies used in flow cytomery analysis of PBMCs.

Antibody	Clone	Conjugation	Supplier
CD45	2D1	APC-H7	BD
CD3	OKT3	FITC<br>BV711	BioLegend
CD8	SK1	BV510	BD
CD4	OKT4	BV605	BioLegend
Gzmb	QA16A02	APC	BioLegend
CD57	NK-1	PE-CF594	BD
IFNG	4S.B3	FITC	BioLegend
HLADR	L243	BV570	BioLegend
CD56	B159	PE-Cy5	BD
CD57	NK-1	PE-CF594	BD
CXCR3	G025H7	PE-Cy7	BioLegend
CXCR5	L138D7	BV650	BioLegend
CD16	3G8	BV570	BioLegend
CD14	HCD14	FITC	BioLegend
CD19	HIB19	BV650	BioLegend
CD10	HI10a	PE-CF594	BD

Table S5. List of primary antibodies used in IHC and mIF studies.

Target	Dilution	Catalog number	Supplier
CD68	1:1000	ab314474	Abcam
CD56	1:100	156R-94	Cell Marque
CD8	1:300	M7103	Dako
CD4	1:400	ab133616	Abcam
PD-1	1:150	LSB12784	LSBio
CD45	1:100	M0701	Dako
CD20	1:200	MS-340	ThermoFisher
PD-L1	1:200	13684	Cell Signaling Techonology
Pan cytokeratin	1:200	ab9377	Abcam
Hexon	1:1000	ab1056	Millipore

Table S6. Adverse events reported according to the Common Terminology Criteria for Adverse Events (CTCAE) v5.0. Clinician-reported events were mapped to the corresponding CTCAE terms, exceptions were kept as reported when no suitable CTCAE term existed (e.g. subfebrile body temperature).

TILT-123 related adverse event	Number of treatment-related adverse events, n (% of all AEs)	Grade 1	Grade 2	Grade 3	Number of unique patients
Chills	14 (31.8)	11 (25)	3 (6.8)	0	6/6 (100)
Fever	10 (22.7)	5 (11.4)	4 (9.1)	1 (2.3)	4/6 (66.7)
Fatigue	4 (9.1)	3 (6.8)	1 (2.3)	0	3/6 (50)
Flu-like symptoms	1 (2.3)	1 (2.3)	0	0	1/6 (16.7)
Subfebrile body temperature	1 (2.3)	1 (2.3)	0	0	1/6 (16.7)
Diarrhea	1 (2.3)	1 (2.3)	0	0	1/6 (16.7)
Headache	1 (2.3)	1 (2.3)	0	0	1/6 (16.7)
Rhinitis	1 (2.3)	1 (2.3)	0	0	1/6 (16.7)
Syncope	1 (2.3)	0	0	1 (2.3)	1/6 (16.7)
Alanine aminotransferase increased	2 (4.5)	2 (4.5)	0	0	2/6 (33.3)
Alkaline phosphatase increased	1 (2.3)	1 (2.3)	0	0	1/6 (16.7)
Aspartate aminotransferase increased	2 (4.5)	2 (4.5)	0	0	2/6 (33.3)
Blood bilirubin increased	1 (2.3)	1 (2.3)	0	0	1/6 (16.7)
Lymphocyte count decreased	4 (9.1)	0	2 (4.5)	2 (4.5)	2/6 (33.3)
Total	**44**	**30**	**10**	**4**	

Table S7. Adverse events reported according to the CTCAE v5.0. Clinician-reported events were mapped to the corresponding CTCAE terms, exceptions were kept as reported when no suitable CTCAE term existed (e.g. subfebrile body temperature, pain at biopsy site) or exact subtype was not specified (e.g. tachycardia). Related events (e.g. shingles, shingle pain, post-herpetic pain) were grouped under the single CTCAE term 'Shingles/herpes zoster' for consistency.

Adverse Event	Grade 1	Grade 2	Grade 3	Grade 4	Grade 5	Total
Chills	11	3	0	0	0	14
Cough	3	0	0	0	0	3
Fever	5	4	1	0	0	10
Rhinitis	2	0	0	0	0	2
Subfebrile body temperature	1	0	0	0	0	1
Fatigue	3	2	0	0	0	5
Flu-like symptoms	1	0	0	0	0	1
Syncope	0	0	1	0	0	1
Headache	2	0	0	0	0	2
Sinusitis	0	1	0	0	0	1
Nausea	2	0	0	0	0	2
Anorexia	2	0	0	0	0	2
Ear pain	2	0	0	0	0	2
Constipation	0	1	0	0	0	1
Diarrhea	1	0	0	0	0	1
Dyspepsia	0	1	0	0	0	1
Dysphagia	1	0	0	0	0	1
Hypertension	0	1	0	0	0	1
Tachycardia	1	0	0	0	0	1
Superficial thromboflebitis	1	0	0	0	0	1
ALT increased	2	0	0	0	0	2
ALP increased	1	0	0	0	0	1
AST increased	2	1	0	0	0	3
Blood bilirubin increased	1	0	0	0	0	1
Lymphocyte count decreased	0	2	2	0	0	4
Myalgia	2	0	0	0	0	2
Abdominal pain	1	2	1	0	0	4
Pain in biopsy site	1	1	0	0	0	2
Fall	1	0	0	0	0	1
Eczema	1	0	0	0	0	1
Pleural effusion	0	1	0	0	0	1
Shingles/herpes zoster	4	2	0	0	0	6

Table S8

Source data for all figures and tables are provided in a separate Excel file titled 'Table S8'

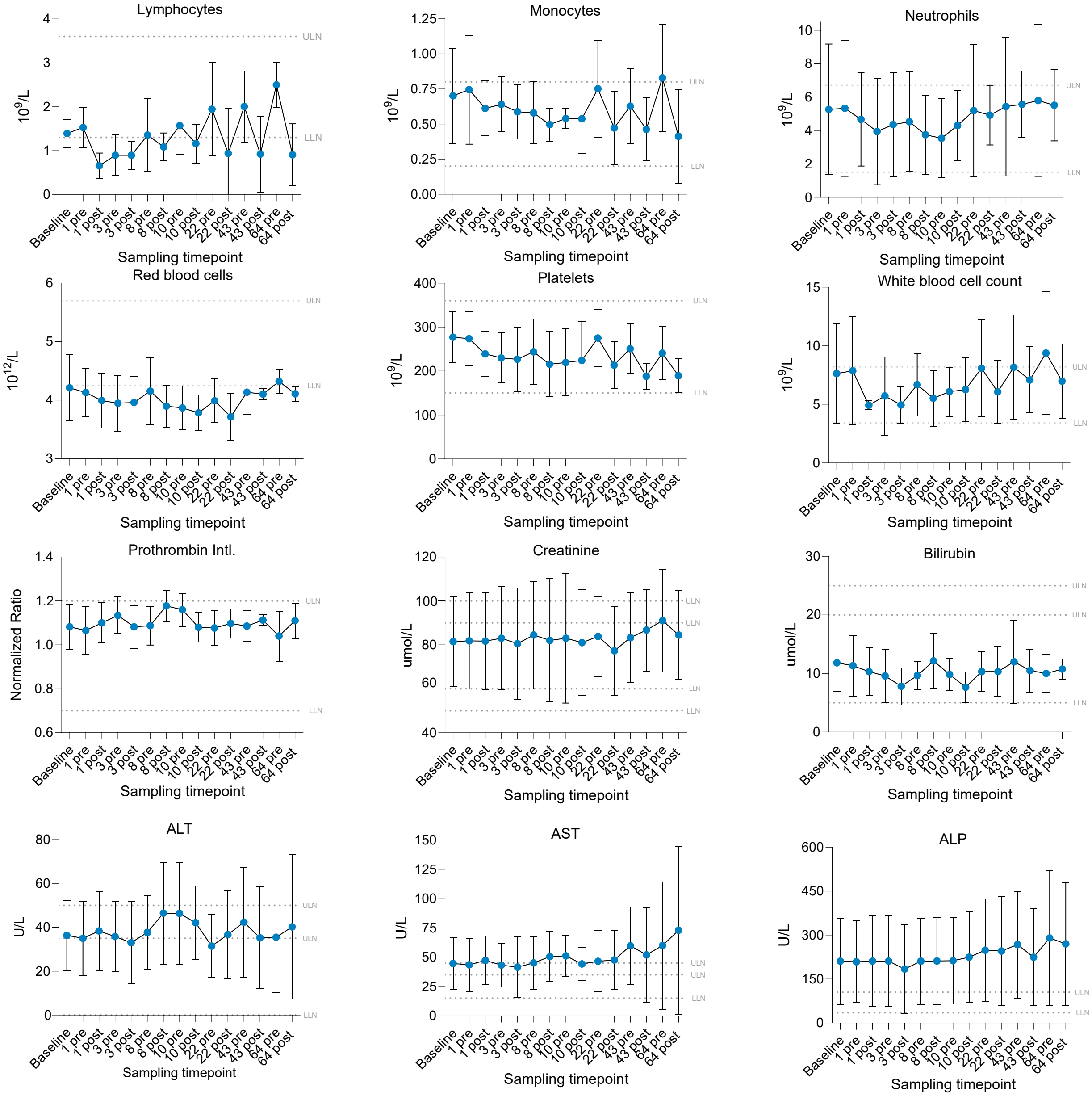

Figure S1. Summary of clinical hematological values throughout the trial. Data are presented as mean ± SD. ULN = upper limit of normal, LLN = lower limit of normal. Baseline to day 43 pre-dosing n = 5-6 patients, day 43 post-dosing to day 64 n = 4 patients.

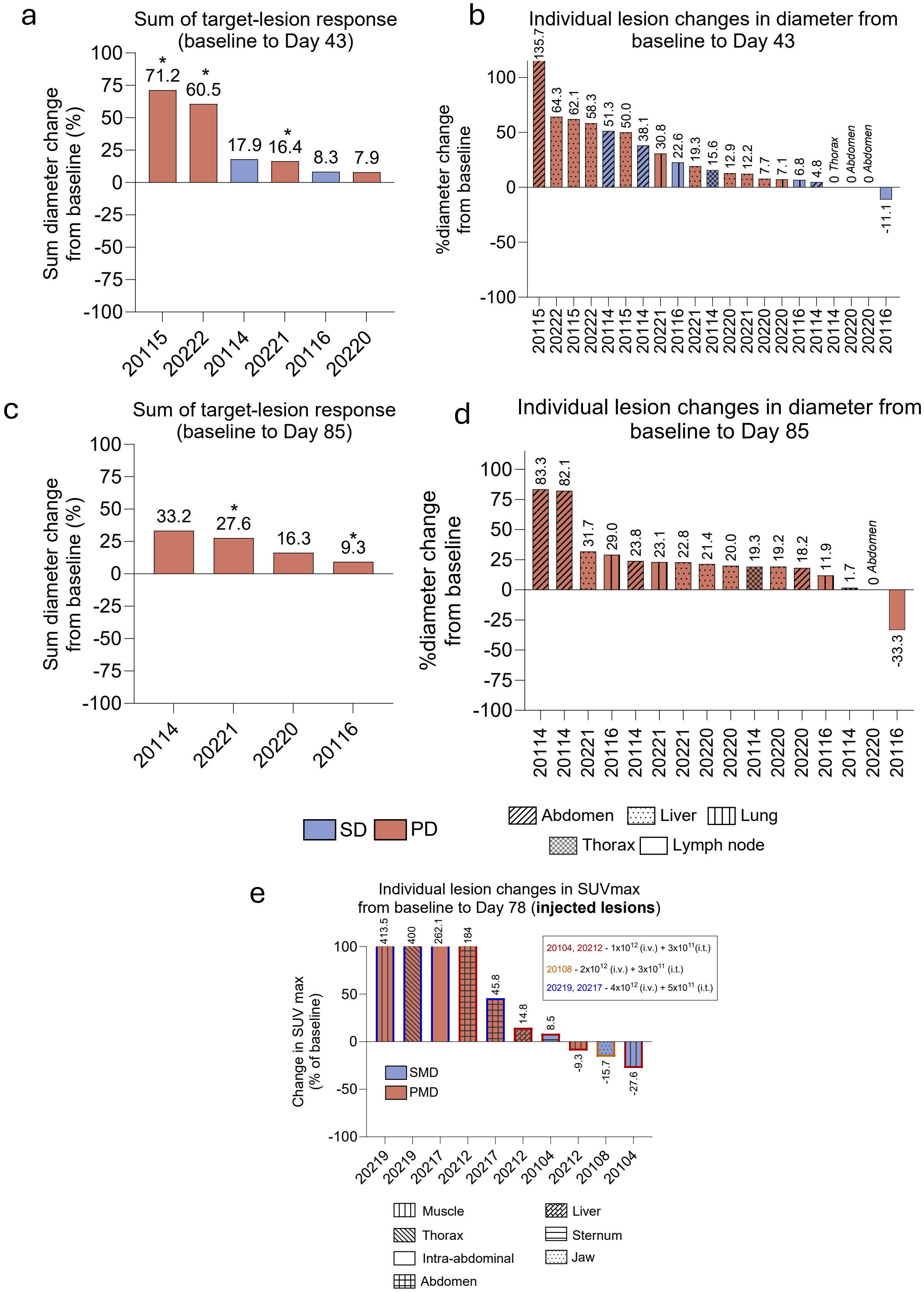

Figure S2. CT and PET-based evluation of target lesions responses across cohorts. a-d) Evaluation of target lesions using CT imaging. * Indicates development of new lesions. SD= stable disease, PD = progressive metabolic disease. e) Individual lesions changes evaluated by PET imaging from patients treated in historical i.t. + i.v. cohorts. SMD = stable metabolic disease, PMD = progressive metabolic disease.

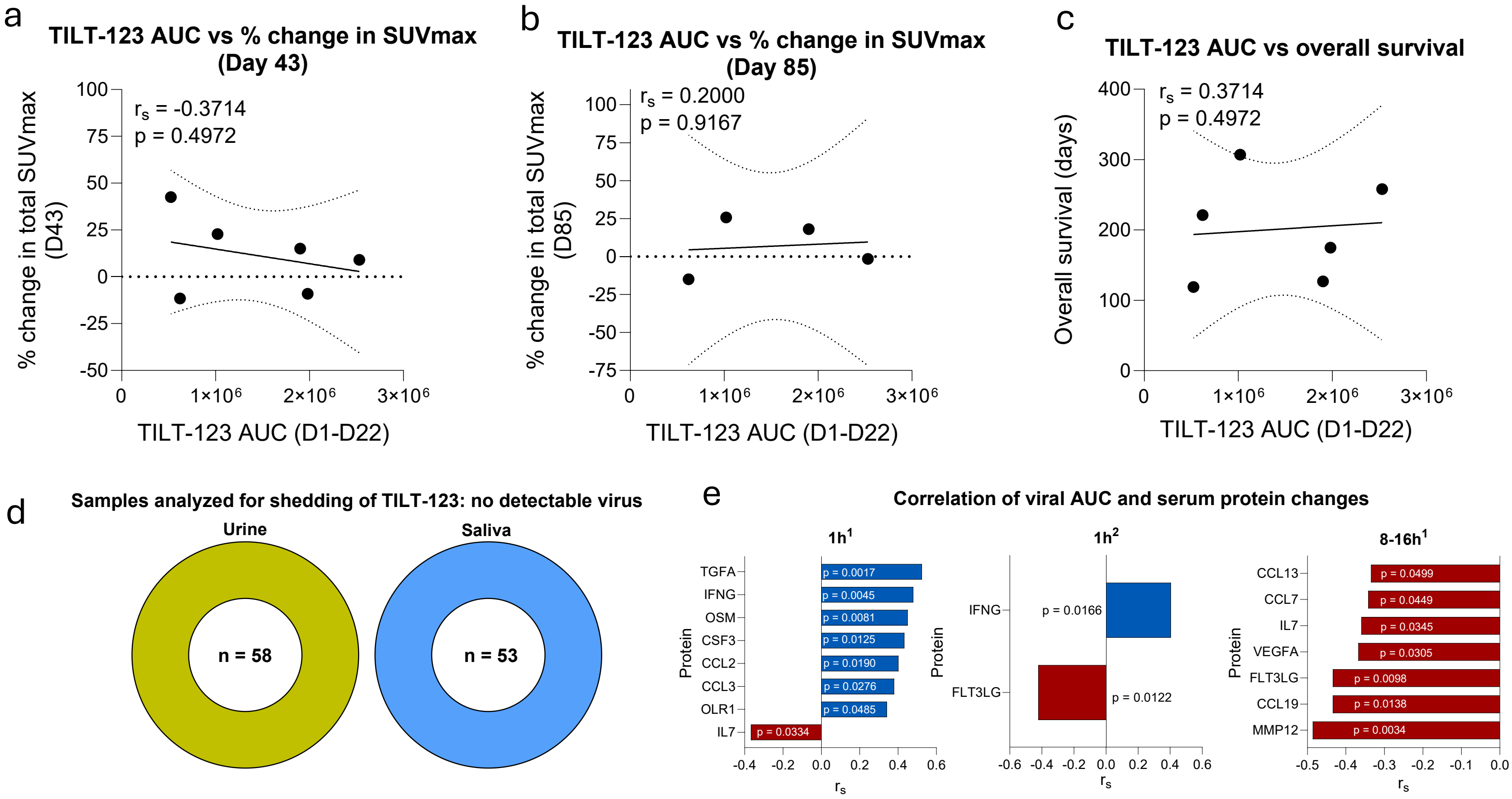

Figure S3. TILT-123 exposure in relation to clinical and proteomic changes, with a summary of shedding data. a-b) Spearman correlation between percentage change in total SUVmax at Day 43 (a) and Day 85 (b) and TILT-123 AUC from Days 1-22. c) Spearman correlation between OS and TILT-123 AUC from Days 1-22. d) Summary of shedding samples analyzed for presence of TILT-123. No virus levels surpassing the LLOQ of 4380 VP/mL were detected. e) Spearman correlation between TILT-123 AUC and serum protein changes post-treatment. Viral DNA AUC was calculated for each treatment day, and correlations were performed seperately for each protein and timepoint.

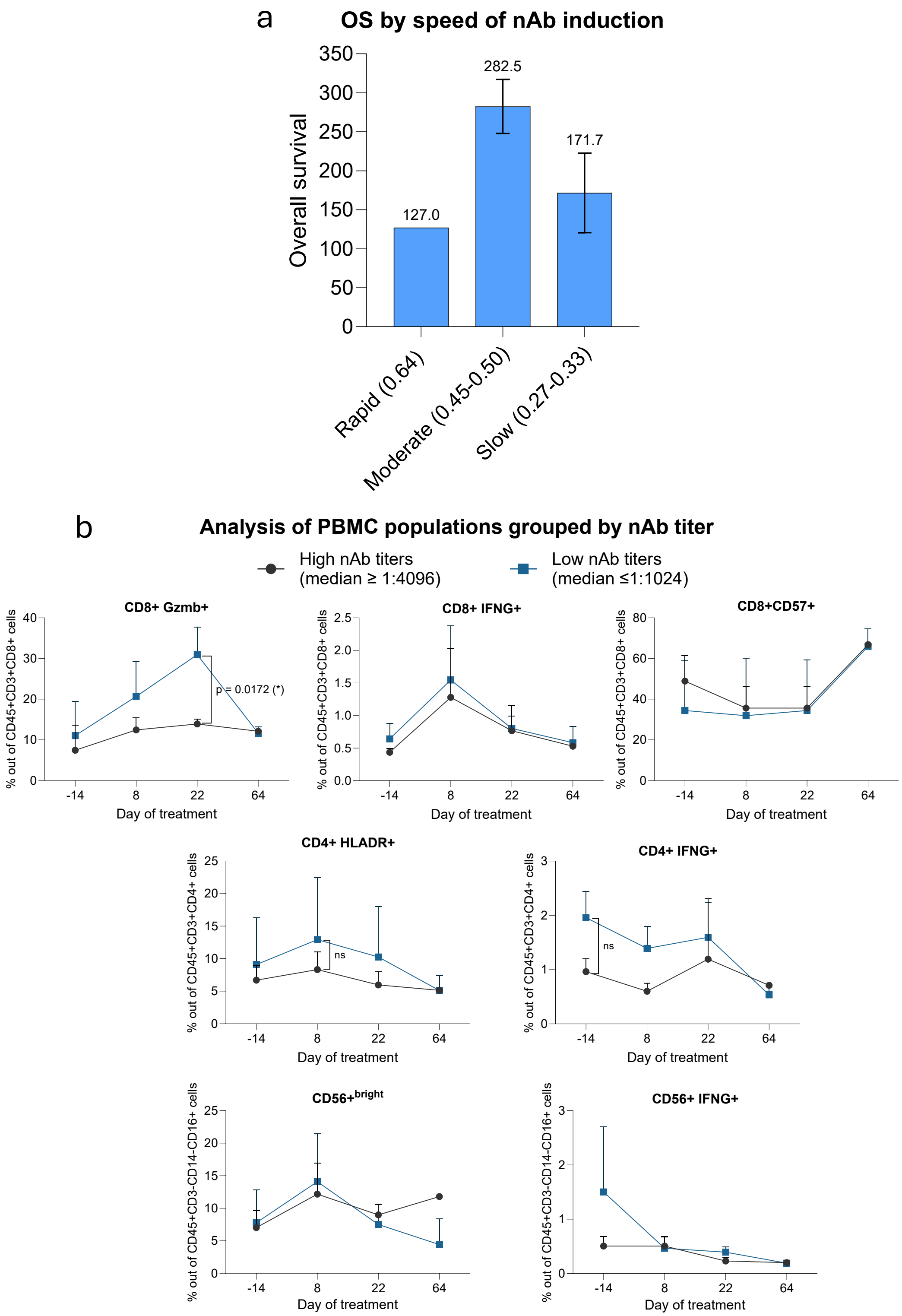

Figure S4. Characterization of nAb kinetics and immune parameters. a) Patient overall survival grouped by speed of nAb induction. Speed of nAb induction was calculated as the log2-fold change from baseline to peak titer divided by the number of days to reach the peak. b) Analysis of PBMC populations grouped by median nAb titer achieved while on treatment. Data are shown as mean ± SD.

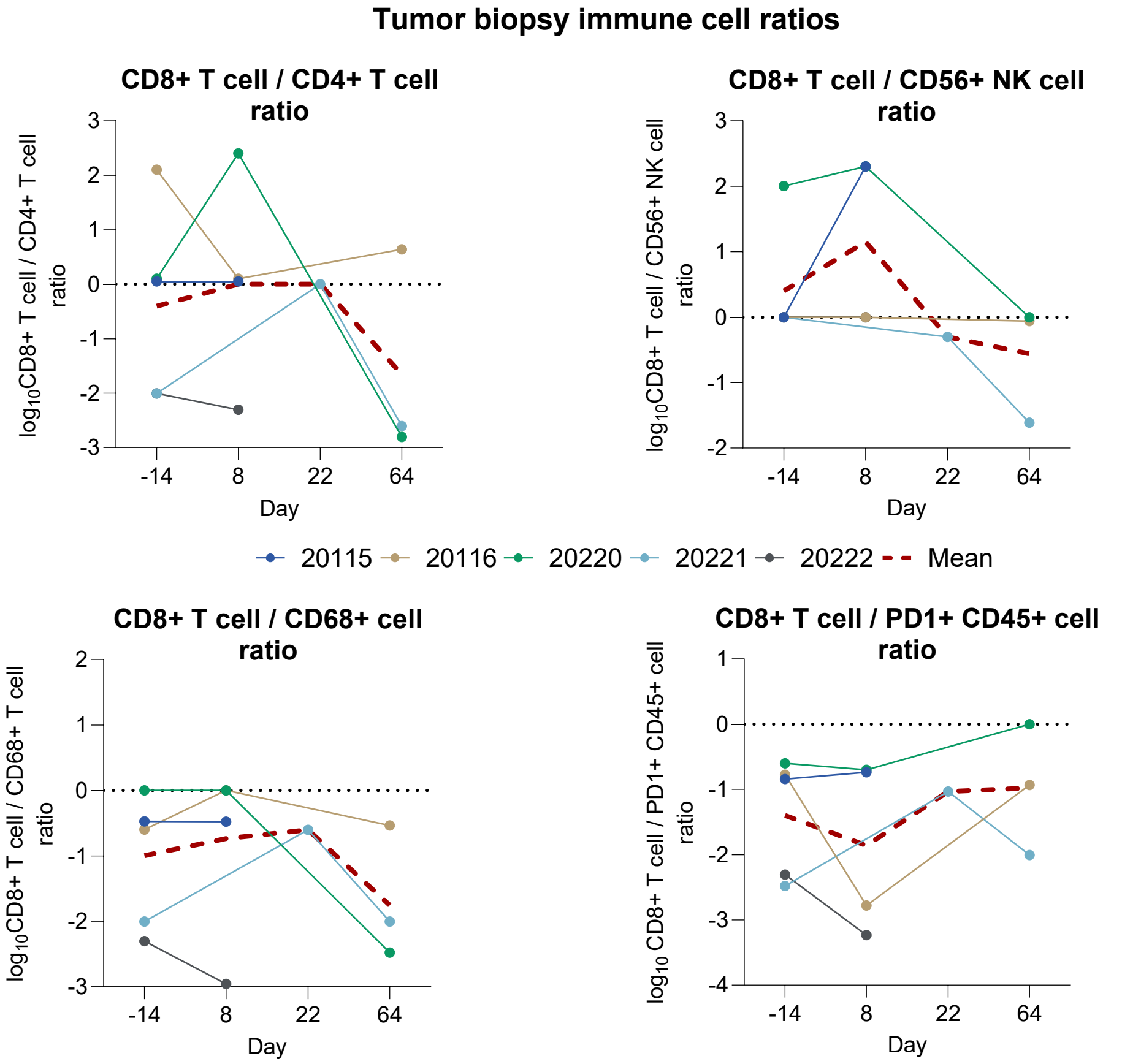

Figure S5. Immune cell ratio analysis in patient tumor biopsies. Individual lines indicate patient biopsies taken pre-treatment (Day -14) and post-treatment (Days 8, 22, and 64). Log_{10} ratios of immune cell populations are shown, where positive values indicate the numerator is more abundant than the denominator, negative values indicate the numerator is less abundant, and zero indicates an equal proportion of both populations. A pseudocount (0.01) was added to all values before to avoid division by zero.
