# Supplementary material for: Fully intravenous split-dose administration of the oncolytic adenovirus TILT-123 in advanced solid tumors
Source: Mol Ther. 2026 May 13;34(8):4670–86. doi: 10.1016/j.ymthe.2026.05.007 (PMC13464138; doi:10.1016/j.ymthe.2026.05.007)
Supplement: Supplementary file 2 [file mmc3.pdf]

Molecular Therapy
Original Article

Fully intravenous split-dose administration of the oncolytic adenovirus TILT-123 in advanced solid tumors

Elise Jirovec,[1,11] Katriina J. Jalkanen,[2,11] Dafne C.A. Quixabeira,[1,3] James H.A. Clubb,[1,3] Tuomo Alanko,[4] Tatiana V. Kudling,[1,3] Victor Arias,[1] Santeri A. Pakola,[1] Susanna Grönberg-Vähä-Koskela,[1,2] Mirte van der Heijden,[1] Anniina Färkkilä,[5,6,7] Juha Kononen,[4] Jorma Sormunen,[4] Jukka Kemppainen,[4,8] Maria Hellesuo,[4] Lyna Haybout,[1,3] Claudia Kistler,[3] Suvi Sorsa,[1,3] Riikka Havunen,[1,3] Joao M. Santos,[1,3] Anna Kanerva,[1,9] Victor Cervera-Carrascon,[1,3] Otto Hemminki,[1,10] and Akseli Hemminki[1,2,3]

[1]Cancer Gene Therapy Group, Translational Immunology Research Program, University of Helsinki, Helsinki, Finland; [2]Comprehensive Cancer Center, HUS Helsinki University Hospital, Helsinki, Finland; [3]TILT Biotherapeutics Oy, Helsinki, Finland; [4]Docrates Cancer Center, Helsinki, Finland; [5]Research Programs in Systems Oncology, Faculty of Medicine, University of Helsinki, Helsinki, Finland; [6]Institute for Molecular Medicine Finland, Helsinki Institute Sciences, University of Helsinki, Helsinki, Finland; [7]Clinical Trials Unit, Department of Oncology & Department of Obstetrics and Gynecology, Helsinki University Hospital, Helsinki, Finland; [8]Department of Clinical Physiology and Nuclear Medicine, Turku University Hospital, Turku, Finland; [9]Department of Gynecology and Obstetrics, Helsinki University Hospital, Helsinki, Finland; [10]Department of Urology, Helsinki University Hospital, Helsinki, Finland

TILT-123 (Ad5/3-E2F-d24-hTNFα-IRES-hIL2, igrelimogene litadenorepvec) is a chimeric oncolytic adenovirus engineered to selectively replicate in tumor cells and express tumor necrosis factor (TNF) and interleukin-2 (IL-2). While oncolytic viruses are commonly administered intratumorally, this approach often restricts practical applicability. Intravenous delivery is less invasive and offers a more practical alternative; however, systemic immune barriers challenge effective tumor targeting. In a cohort of a phase 1 clinical trial, six patients with advanced solid tumors who had exhausted standard treatments received two intravenous doses of TILT-123 (split-dose), per treatment day across seven cycles. Safety was the primary objective, while efficacy outcomes were exploratory and assessed using positron emission tomography/-computed tomography (PET/CT) imaging, along with overall survival. Split-dosing was safe, with chills, fever, fatigue, and lymphocyte reduction being the most common treatment-related events. In imaging, the disease control rate was 83.3% according to PET criteria and 33.3% by RECIST 1.1. The median overall survival was 198 days. Split-dosing increased the area under the curve of TILT-123 and amplified systemic cytokine responses. Proteomic analysis of serum and neutralizing antibody profiling indicated that enhanced humoral immune activity was associated with shorter overall survival. Post-treatment biopsies confirmed virus presence and alterations in immune cell composition. These findings support further evaluation of intravenous TILT-123 administration in clinical trials.

INTRODUCTION

The emergence of immunotherapies, particularly immune checkpoint inhibitors (ICIs), have transformed the treatment of many solid tumors.[1] However, response rates remain modest, with a minority of patients achieving long-term benefit, highlighting the need for innovative treatments.[2–4] Oncolytic viruses (OVs) are a class of therapeutic agents that selectively replicate in and lyse tumor cells, while activating adaptive and innate immune responses.[5] Additionally, OVs can be genetically modified to carry transgenes, which are expressed and released locally from infected tumor cells during viral replication. These transgene-encoded proteins can further enhance antitumor activity by, for example, increasing immune cell trafficking to the tumor microenvironment (TME).[6] OVs may overcome some of the limitations of T cell therapies including ICIs, such as insufficient intratumoral (i.t.) immune cell infiltration and an immunosuppressive TME. Through tumor lysis and release of tumor-associated antigens, along with danger- and pathogen-associated molecular patterns, OVs can transform an immunologically "cold" tumor into an inflamed, immune-infiltrated landscape primed for antitumor immune activation.[7]

Currently, the only FDA-approved OV for clinical use, talimogene laherparepvec (T-VEC), is indicated for unresectable metastatic melanoma and administered via i.t. injection. While effective for accessible lesions, i.t. delivery is limited by its invasiveness and practicality, necessitating image-guided injection and specialized personnel, and it poses potential risks such as bleeding or

Received 1 December 2025; accepted 5 May 2026;
https://doi.org/10.1016/j.ymthe.2026.05.007.

[11]These authors contributed equally

Correspondence: Akseli Hemminki, Cancer Gene Therapy Group, Translational Immunology Research Program, University of Helsinki, Haartmaninkatu 8, 00290 Helsinki, Finland.
E-mail: akseli.hemminki@helsinki.fi

Published by Elsevier Inc. on behalf of The American Society of Gene and Cell Therapy.
This is an open access article under the CC BY license (http://creativecommons.org/licenses/by/4.0/).

Table 1. Patient demographics and clinical characteristics

Patient ID	Age, y	Sex	ECOG status	Cancer type	Stage	Best overall response RECIST 1.1	Best overall response iRECIST	Best overall PET response	No. of previous systemic treatments	OS, days
20114	62	F	1	Liposarcoma	IV	SD	iSD	SMD	2	307
20115	49	F	0	Gastric adenocarcinoma	IV	PD	iUPD	PMD	3	119
20116	59	M	1	PDAC	IV	SD	iSD	MMR	4	221
20220	66	M	1	Rectal carcinoma	IV	PD	iUPD	SMD	4	258
20221	63	M	0	Rectal carcinoma	IV	PD	iUPD	SMD	6	127
20222	71	M	1	Rectal carcinoma	IV	PD	iUPD	SMD	6	175

ECOG, Eastern Cooperative Oncology Group; iSD, immune stable disease; iUPD, unconfirmed progressive disease; MMR, minor metabolic response; OS, overall survival; PD, progressive disease; PDAC, pancreatic ductal adenocarcinoma; PET, positron emission tomography; PMD, progressive metabolic disease; SD, stable disease; SMD, stable metabolic disease.

injection-site discomfort.[8,9] In contrast, intravenous (i.v.) delivery offers a systemic, less invasive, and more easily standardized route of administration that can target both primary and metastatic tumor sites, thereby broadening therapeutic reach.[10] Despite clear logistical and clinical advantages, i.v. delivery of OVs faces significant challenges, primarily due to the innate and adaptive antiviral immune defense of the host, which might neutralize the virus and limit its ability to reach tumors in sufficient quantities.[11] In particular, the liver acts as a major sink for i.v.-administered adenovirus.[12]

Preclinical studies in mouse models have demonstrated that >90% of systemically injected oncolytic adenovirus is rapidly sequestered by the liver, as soon as 11 s post-injection.[13,14] Resident macrophages of the liver, known as Kupffer cells (KCs), play a central role in this process. KCs act as the first line of defense against pathogens and constitute 80%–90% of the body's tissue resident macrophage population, representing a significant barrier to effective systemic delivery of OVs.[15] Interestingly, multiple preclinical studies have demonstrated that KCs may be depleted or undergo necrosis by high viral doses or agents such as clodronate liposomes and lipid emulsions, resulting in significantly increased circulating viral titers upon subsequent dosing.[16–18] Based on these findings, we hypothesized that a split-dosing strategy, where the virus is administered in two i.v. injections per treatment day, could enhance systemic bioavailability. The rationale is that the initial dose may temporarily saturate or impair KC function, thereby allowing increased viral circulation and tumor transduction after the second injection.

In this study, we tested this strategy using TILT-123 (Ad5/3-E2F-d24-hTNFα-IRES-hIL2, igrelimogene litadenorepvec) in patients with advanced solid tumors. TILT-123 is a chimeric serotype 5/3 oncolytic adenovirus, engineered to selectively replicate in tumor cells and express tumor necrosis factor (TNF) and interleukin-2 (IL-2), chosen for their superior antitumor efficacy and immune cell recruitment compared with other cytokines in preclinical studies.[19,20] The 5/3 capsid may confer an advantage for i.v. delivery by circumventing preexisting immunity to wild-type adenovirus.[21] Furthermore, dual-selectivity devices prevent virus replication in normal tissues, allowing the safe use of high doses.

This article reports results from a cohort of six patients enrolled in the phase 1 clinical trial TUNIMO (ClinicalTrials.gov: NCT04695327) treated with i.v. TILT-123 in a split-dose regimen. We focus on evaluating the safety, bioavailability, immune responses, and overall treatment outcomes associated with this approach.

RESULTS

Patient demographics

Six patients with a median age of 62.5 (49–71) years were treated in cohort 7 of the TUNIMO clinical trial. Half of the patients had a diagnosis of rectal adenocarcinoma, and the other diagnoses were liposarcoma, gastric adenocarcinoma, and pancreatic ductal adenocarcinoma (PDAC). All patients had stage IV disease and had received a median of four previous lines of systemic therapy (Table 1).

Safety of fully i.v. TILT-123

The fully i.v. regimen was well tolerated, with a favorable safety profile. The most common treatment-related adverse events (AEs) included chills (14/44; 31.8% of all AEs), fever (10/44; 22.7%), fatigue (4/44; 9.1%), and lymphocyte count decrease (4/44; 9.1%). Chills were reported in all patients. Four grade 3 AEs occurred: fever (1/44; 2.3%); syncope (1/44; 2.3%) following the second TILT-123 dose; and lymphocyte count decrease (2/44; 4.5%), which had normalized by the next blood sampling. Transient alanine aminotransferase and aspartate aminotransferase elevations occurred in 33.3% (2/6) of patients and resolved without intervention. TILT-123-related AEs are summarized in Table S6. All AEs recorded during the trial, regardless of relation to treatment, are found in Table S7. Trends in hematology and clinical chemistry values are shown in Figure S1.

Patient outcomes

Given the limited cohort size and heterogeneous tumor types, efficacy outcomes are presented as exploratory observations. All

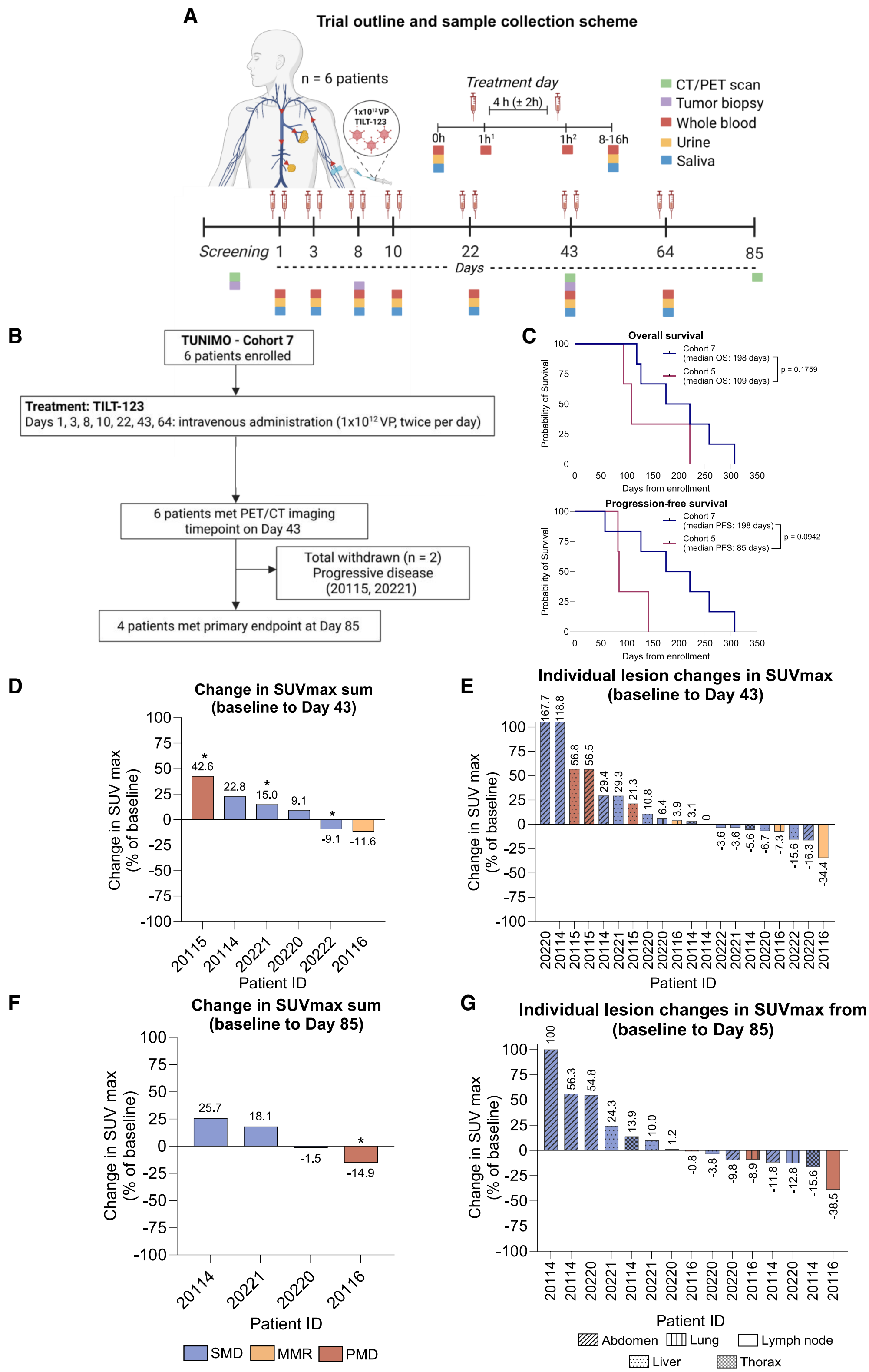

(legend on next page)

patients underwent positron emission tomography (PET) and computed tomography (CT) imaging on day 43, and 66.7% (4/6) of the patients met the primary endpoint of safety on day 85 (Figure 1B). Median overall survival (OS) and progression-free survival (PFS) of patients were 198 days in the split-dose group, compared with 109 and 85 days, respectively, in the single-dose-equivalent i.v. plus subsequent i.t. TILT-123 cohort (Figure 1C).

On day 43, PET-based evaluation demonstrated stable metabolic disease in 66.7% (4/6) of patients, one minor metabolic response (1/6), and one progressive metabolic disease (1/6) (Figure 1D). Based on CT imaging assessed by RECIST 1.1 criteria, 33.3% (2/6) of patients demonstrated stable disease (Figure S2A). Overall, 40% (8/20) of target lesions showed decreased maximum standardized uptake value (SUVmax), with half of these being located in the liver (Figure 1E). CT-based individual lesion response is shown in Figure S2B. Two patients discontinued treatment on day 43 due to suspicion of disease progression.

At the primary endpoint, 75% (3/4) of evaluable patients maintained stable metabolic disease by PET criteria (Figure 1F), while all had progressive disease by CT (Figure S2C). On day 85 imaging, 46.7% (7/15) of target lesions had reduced SUVmax, with the largest decrease observed in a lymph node lesion (Figure 1G). CT-based individual lesion response on day 85 is shown in Figure S2D. Compared with other cohorts, where patients received combined i.v. and i.t. TILT-123, primary endpoint imaging showed a decrease in SUVmax values in 30% (3/10) of injected lesions (Figure S2E). Based on the best response observed across all imaging time points, disease control was achieved in 83.3% (5/6) of patients by PET and 33.3% (2/6) of patients by CT.

Circulating TILT-123 associates with fever, infectious particles, and pro-inflammatory responses

TILT-123 kinetics were consistent across patients, with viral DNA levels reaching their highest observed values 1 h after the first or second dose and declining by the final sampling time point (Figure 2A). Split-dosing did not significantly prolong viral circulation or increase viral levels in blood at individual time points compared with single i.v. dosing (Figure 2B). However, the total viral exposure as measured by area under the curve (AUC) was approximately doubled in split-dose patients (1.29×10^6 vs. 6.62×10^5; Figure 2C). However, systemic exposure to TILT-123, as measured by AUC, did not correlate with changes in total SUVmax or with OS (Figures S3A–S3C). TILT-123 was not detected above the quantification limit in urine or saliva (Figure S3D). TILT-123 was generally undetectable in pre-treatment blood on subsequent visits, except for patient 20220 on day 3, who presented with fever and had TILT-123 treatment omitted (Figure 2D).

Prompted by this observation, we examined whether circulating TILT-123 DNA in the blood was associated with fever and found higher viral DNA levels 1 h after the first dose in febrile patients ($p = 0.0495$), with a similar but not statistically significant trend 1 h after the second dose (Figure 2E). To explore the relationship between dosing frequency and AEs, we analyzed AE distribution by visit day and observed the highest number of AEs on day 8, with most related to influenza-like symptoms and changes in liver function (Figure 2F). We performed a cell-based infectivity assay on a subset of post-treatment blood samples with high TILT-123 DNA levels, as qPCR indicates the presence of viral DNA, not infectivity. Infectious TILT-123 particles were identified in 2 of 15 samples, with positive samples from 2 different patients, providing proof-of-concept that qPCR-detected virus in blood can infect tumor cells. Representative images show green foci corresponding to infectious TILT-123 (Figure 2G). To further investigate the impact of the presence of TILT-123 on systemic immune responses, we examined correlations between viral DNA levels and fold changes in serum proteins at matched time points (Figure 2H). Several proteins, including IL-17F, C-C motif chemokine ligand 3 (CCL3), interferon-γ (IFN-γ), IL-27, cerebrospinal fluid 2 (CSF2), IL-2, and IL-6, showed positive correlations, indicating that elevated viral DNA levels may be linked to pro-inflammatory responses. We next evaluated whether systemic exposure to TILT-123, measured as the AUC of TILT-123 DNA, correlated with changes in serum protein levels at different post-treatment time points (Figure S3E). One hour after the first dose, most proteins positively correlated with TILT-123, indicating an early pro-inflammatory response proportional to viral exposure, while 8–16 h after the first dose, correlations were uniformly negative, potentially reflecting cytokine clearance following peak viral exposure.

Sequential TILT-123 doses amplify cytokine responses and reveal serum biomarkers associated with survival

Temporal profiles of selected cytokines are shown in Figure 3A. Concentrations of TNF and IL-2, which are TILT-123-encoded proteins but also endogenously expressed, were typically highest either 1 h after the second dose or at the 8- to 16-h time points. IFN-γ and C-X-C motif chemokine ligand 10 (CXCL10), markers of antiviral response and immune cell recruitment, also showed dynamic changes: IFN-γ peaked on day 1, with a secondary rise on day 8, while CXCL10 spiked at later time points and showed elevated pre-treatment levels on days 3, 8, and 10, coinciding with intensive dosing days.

Figure 1. Trial overview and patient outcomes

(A) Schematic of trial outline indicating treatment days, imaging, and sample collection time points. (B) Outline of patients ($n = 6$) treated in cohort 7 of TUNIMO. (C) Kaplan-Meier survival curves comparing overall survival (OS) and progression-free survival of dose-equivalent single i.v. TILT-123-treated patients versus split-dose-treated patients. Comparison of survival curves was performed using log rank test. (D–G) Evaluation of target lesions using PET imaging. The asterisk indicates the development of new lesions. MMR, minor metabolic response; PMD, progressive metabolic disease; SMD, stable metabolic disease. Day 85 20114 abdominal lesion had an increase of SUVmax from 0 to 1.9; nominal value of 100% assigned for visualization purposes only; actual percent change is undefined.

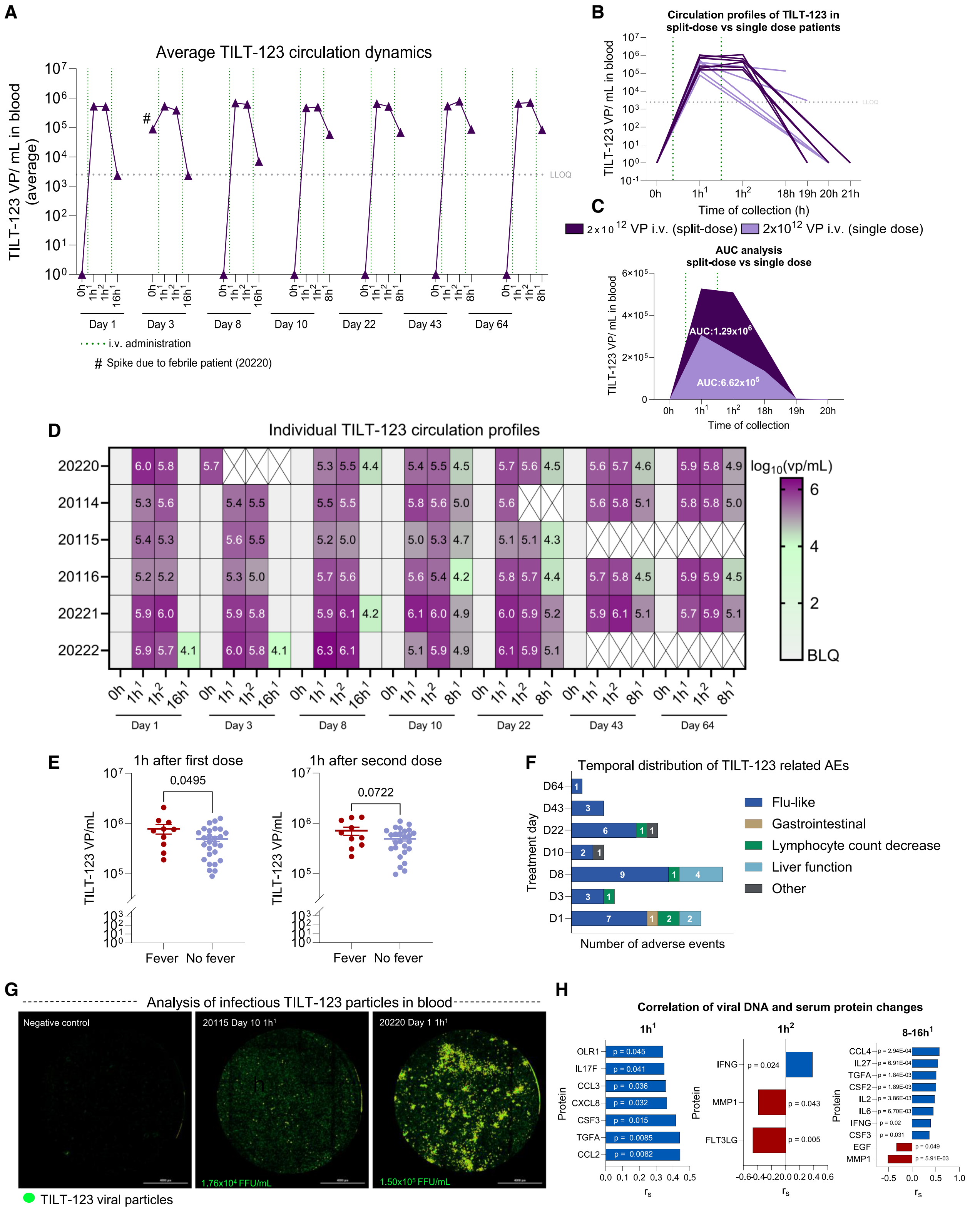

(legend on next page)

To explore the effects of each TILT-123 dose, we compared serum profiles after dosing to baseline (Figure 3B). Interestingly, the first dose caused a downregulation of proteins related to innate immunity, followed by an upregulation of pro-inflammatory proteins 1 h after the second dose and 8–16 h after the first dose. Direct comparison of 1-h time points after each dose (Figure 3C) showed significant upregulation of proteins following the second dose, indicating an enhanced systemic immune response involving cytokine production, granulocyte migration, and JAK-STAT signaling (Figure 3D). Additionally, split-dose i.v. administration induced significantly higher fold changes in CCL8, EGF, CXCL10, and IFN-γ concentrations compared to single-dose i.v. (Figure 3E). Serum protein levels were assessed for correlation with OS to identify potential markers associated with the therapeutic benefit of TILT-123 (Figure 3F). Proteins positively correlated with OS were enriched in pathways such as positive regulation of IL-17 production. In contrast, proteins negatively associated with OS were linked to the humoral immune response and the positive regulation of chemokine production (Figure 3G).

To investigate the relationship between humoral responses and treatment outcome, we examined neutralizing antibody (nAb) titers across the trial (Figure 4A). Titers peaked around day 8 in half of the patients, with two reaching the maximum measurable titer at this time point. Patients with above-median OS had significantly lower nAb titers while on-treatment than those below the median ($p = 0.0028$; Figure 4B). To better explore how the humoral response may negatively affect TILT-123 therapy, we correlated nAb titers and serum proteins on day 8 and found a negative association with several proteins involved in immune cell migration (CSF1, IL-33, OLR1, OSM, TNF, CCL2, and CXCL10) (Figures 4C and 4D). Viral DNA levels post-treatment positively correlated with nAb titers ($p = 0.0001$; Figure 4E). To characterize the dynamics of the speed of nAb responses, we calculated the rate of increase in $\log_2$ titer U/day from baseline to peak titers, where higher values indicate faster nAb induction. nAb induction rates varied substantially across patients, ranging from 0.27 to 0.64 $\log_2$ U/day (Figure S4A). We observed a non-linear pattern between induction rate and OS. Patients were grouped by induction category: the mean OS was 282 days for moderate responders ($n = 2$) and 172 days for slow responders ($n = 3$), while the single rapid responder ($n = 1$) had an OS of 127 days. Given the small sample size, particularly the single patient in the rapid responder group, these observations are descriptive only, and no statistical comparisons were performed.

Given the association between high nAb titers and reduced therapeutic benefit, we next evaluated immune activation in the cellular compartment by profiling peripheral blood mononuclear cells (PBMCs) using flow cytometry (Figure 4F). $CD8^+$ T cells demonstrated increased granzyme B (day 22, $p = 0.0425$) and IFN-γ (day 8, $p = 0.0303$) expression, indicating enhanced cytotoxic function. $CD4^+$ T cells exhibited increased human leukocyte antigen-DR isotype ($p = 0.0392$) and decreased IFN-γ ($p = 0.0392$) expression. Analysis of the innate compartment revealed a significant increase in $CD56^+$ bright natural killer (NK) cells from baseline to day 8 ($p = 0.0408$) and an increase in $CD56^+$ dim cells from baseline to day 64 ($p = 0.0396$), while IFN-γ^+ NK cells showed a non-significant decreasing trend ($p = 0.0881$), suggesting possible modulation of NK activity. $CD19^+$ B cells and $CD10^-$ plasma cells increased significantly by day 8 and then seemed to stabilize to near baseline levels at later time points. When analyzing effector PBMC subsets by median nAb titers, no statistically significant differences were observed, except for granzyme B-expressing $CD8^+$ T cells, which were significantly increased on day 22 in patients with low nAb titers ($p = 0.0172$; Figure S4B).

Analysis of liver macrophage-associated markers after split-dosing

To assess potential KC responses to split-dosing, we analyzed serum markers associated with macrophage and inflammatory activation. Upon activation, KCs and other myeloid cells secrete TNF, IL-1, IL-6, CXCL1–3, CXCL8, and CCL2–4, which contribute to complement activation and the initiation of inflammatory responses.[22] TNF, IL-6, CXCL8, and CCL2 levels showed a trend toward a slight decrease after the first dose and an increase after the second dose and at 8–16 h, depending on the treatment day (Figure 5A). CCL3 appeared to increase after the second dose on later treatment days and then dropped by 8–16 h post-treatment. Since these markers are not KC specific, we measured soluble CD163 (sCD163) serum levels. sCD163, a more specific marker

Figure 2. TILT-123 bioavailability, infectivity, and clinical correlations

(A) Average levels of circulating TILT-123 DNA in whole blood pre- and post-treatment, as detected by qPCR. $1h^1$, 1 h post-first dose; $1h^2$, 1 h post-second dose, $8h^1$–$16h^1$, 8–16 h post-first dose. Blood was collected at 16 h on days 1, 3, and 8 and at 8 h on subsequent visit days to reduce hospitalization time. The lower limit of quantification (LLOQ) for this assay, based on an input of 100 ng DNA, was 2,500 VP/mL. (B) Individual profiles of circulating TILT-123 levels between split-dose ($n = 6$) and dose-equivalent single-dose treatment groups from clinical trials TUNIMO, PROTA, and TUNINTIL ($n = 8$), as detected by qPCR. (C) Area under the curve analysis of circulating TILT-123 levels in patients receiving the split-i.v. dose or single-i.v. dose. (D) Individual profiles of circulating levels of TILT-123 DNA. Viral particles (VP) per milliliter values were transformed into $\log_{10}$(VP/mL) for data visualization. Time points with unavailable data are indicated by X. BLQ, below the limit of quantification. (E) TILT-123 DNA levels in whole blood 1 h post-dose between patients with and without reported fever. Differences between groups were assessed using unpaired t test. (F) Distribution of TILT-123-related AEs per treatment day grouped by category. AEs recorded between treatment visits were assigned to the most recent treatment day. AEs including chills, fatigue, fever, rhinitis, and subfebrile body temperature were grouped under the influenza-like category. (G) Analysis of whole blood using a cell-based infectivity assay where green foci indicate infectious TILT-123 particles. FFU, focus-forming unit. Cells were stained with rabbit anti-adenovirus type 5 antibody (1:2,000, Abcam) followed by Alexa Fluor 488 goat anti-rabbit immunoglobulin G (1:1,000, Thermo Fisher). Foci were imaged and quantified using a Cytation 10 imaging system (Agilent). White scale bar indicates 4000 μm. (H) Spearman correlation of TILT-123 DNA levels and changes in serum proteins. Changes in serum protein levels from baseline to post-treatment were calculated for each visit day and correlated with matched viral DNA levels in blood.

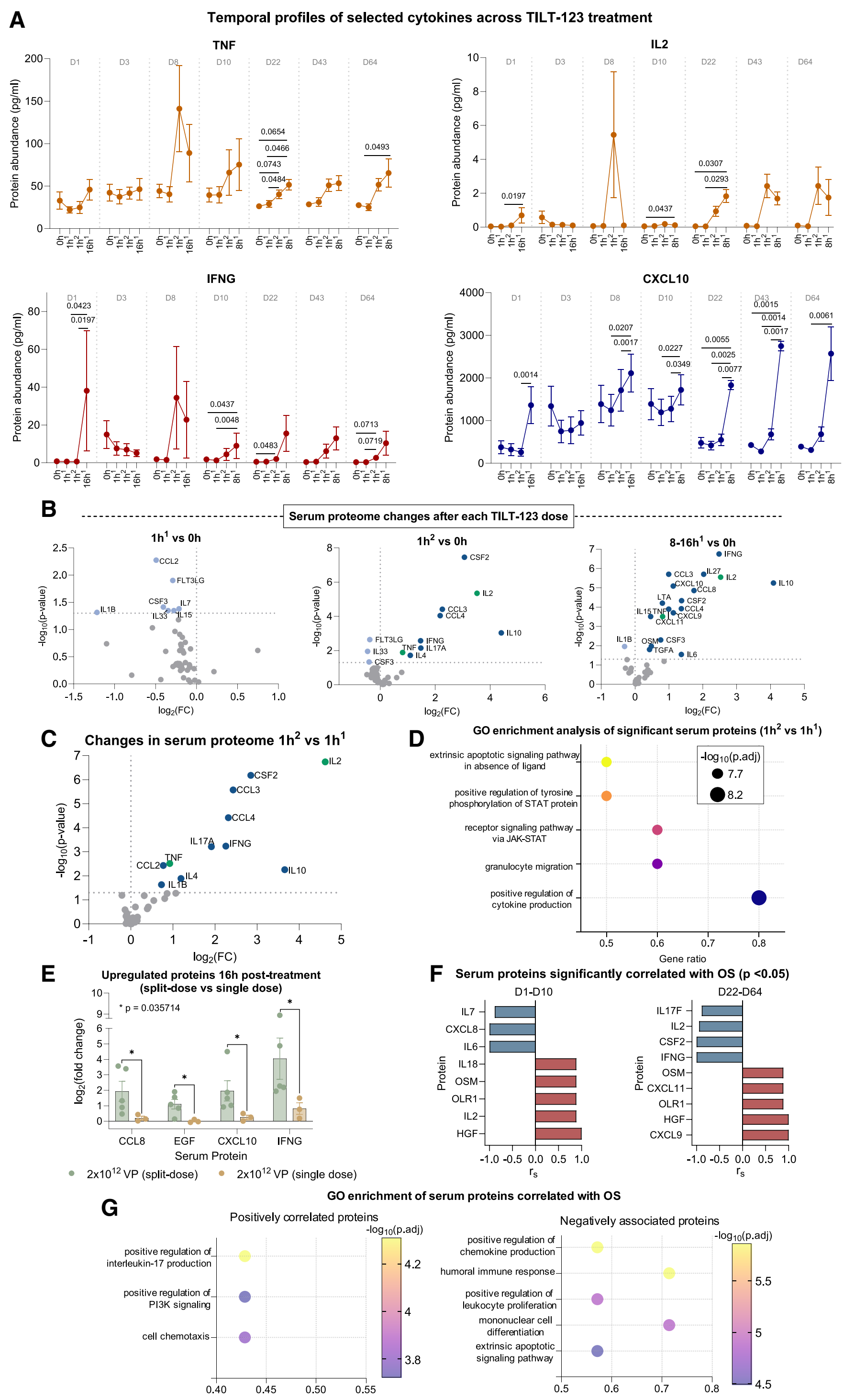

Figure 3. Profiling of treatment-induced serum proteome changes and clinical correlations

(A) Serum proteomic analysis of key cytokines across all time points. Data are presented as mean ± SD. Paired comparisons across time points were evaluated using repeated measures one-way ANOVA or Friedman test with Tukey's or Dunn's multiple comparisons test, depending on the data distribution. p values <0.1 are displayed. Days 1–22, $n = 5–6$, days 43–64, $n = 4–6$. (B) Volcano plots of significantly upregulated serum proteins post-treatment (1 h post-first dose, 1 h post-second dose, and 8–16 h post-first dose) across all treatment days compared to baseline. Mann-Whitney U test was used to assess overall differences in protein levels. Proteins with a p value <0.05

(legend continued on next page)

of macrophage activation, can be detected in the serum and has been associated with liver inflammation and disease.[23] Although not statistically significant, sCD163 levels showed a modest dip after both doses on day 1, returning to baseline by 16 h (Figure 5B). Viral DNA levels were measured 1 h after each dose to assess whether the first dose saturated KCs and increased bioavailability of the subsequent dose. However, no statistically significant differences were observed (Figure 5C).

To investigate the effect of split-dosing on hepatic macrophages, we performed multiplex immunofluorescence (mIF) staining for CD68 in liver biopsies containing tumoral, stromal, and peritumoral areas. CD68 is a marker for macrophages, but it can also be expressed by some subsets of dendritic cells. Representative images show a qualitative increase in $CD68^+$ cells on day 8 of treatment (Figure 5D). Quantification of $CD68^+$ densities in epithelial (Epi^+; tumor) and stromal/peritumoral (Epi^-) compartments indicated interpatient and compartment-specific variability (Figure 5F). In three out of four patients (nos. 20115, 20220, and 20222), epithelial (tumor) regions showed an increase in $CD68^+$ cell infiltration, whereas stromal/peritumoral $CD68^+$ levels remained low and relatively unchanged, suggesting modest trends in macrophage-related markers and spatial distribution following split-dosing.

i.v. TILT-123 results in tumor transduction and immune modulation in the TME

Qualitative increases in $CD4^+$ and $CD8^+$ T cells were seen in post-treatment liver biopsies compared with baseline (Figure 6A). Quantitative mIF analysis showed dynamic fluctuations in $CD4^+$, $CD8^+$, $CD56^+$, and $CD68^+$ cells following treatment (Figure 6B). Notably, patient 20220 exhibited pronounced increases in all four immune cell types on day 43. A statistically significant increase in $CD4^+$ T cells was detected across patients between baseline and day 43 ($p = 0.0179$). Immune checkpoint receptor analysis revealed elevated programmed death ligand-1 (PD-L1) expression on tumor cells post-treatment. Additionally, $PD\text{-}1^+CD45^+$ immune cells were significantly increased by day 8 ($p = 0.0397$), suggesting early immune engagement. Analysis of tumor biopsy immune cell ratios showed that the CD8:CD4 T cell ratio was approximately balanced from baseline through day 22 and decreased by day 64, indicating a relative abundance of $CD4^+$ T cells at the later time point (Figure S5). CD8:CD56 ratios indicated higher $CD8^+$ T cell prevalence at baseline and day 8, shifting toward NK cell predominance at later time points. CD8:CD68 ratios were consistently negative, reflecting a higher abundance of macrophages compared with $CD8^+$ T cells, and $CD8{:}PD1^+CD45^+$ ratios remained negative throughout the trial.

Proteomic analysis of tumor lysates did not yield statistically significant changes at conventional thresholds; however, several proteins were differentially expressed using an exploratory cutoff of $p < 0.2$ (Figure 6C). To assess whether the expression of specific proteins within the tumor were linked to certain immune cells detected by mIF, we performed correlation analyses (Figure 6D). IL-2 protein levels were positively correlated with $CD8^+$ T cell infiltration. Among $CD4^+$ T cells, CXCL12 levels showed a positive correlation, whereas IFNL1 and HAVRC1 levels were negatively correlated. $CD68^+$ cells were negatively correlated with TNFRSF9, LIF, IL-20, and HAVRC1 levels. Finally, $PD1^+CD45^+$ cells were negatively associated with TNFRSF9 levels. Transcriptomic analysis of tumors did not identify any statistically significant changes post-treatment. Exploratory analysis of fold change patterns indicated potential trends toward the upregulation of genes such as *VEGFA*, *PPBP*, and *MX1* and the downregulation of genes such as *MARCO*, *AMBP*, *CCL19*, and *C9*, with some overlapping genes between the two post-treatment time points (Figure 6E).

To evaluate the presence of TILT-123 within tumors, qPCR analysis was performed on tumor homogenates. TILT-123 DNA was detected in liver tumor biopsies from two patients on day 8 (Figure 6F). In the split-dose i.v. cohort, TILT-123 DNA or protein was detected in two of five patients, which is similar to or higher than cohorts receiving hybrid (i.v. + i.t.) TILT-123, indicating that split-dose administration does not compromise tumor delivery (Figure 6G). Immunohistochemistry (IHC) for adenoviral hexon protein confirmed tumor-localized TILT-123 in 1 of 5 evaluable patients, with a representative image from PDAC patient 20220 shown in Figure 6H. Importantly, in the single PDAC patient (no. 20116), PET imaging of a subcarinal lymph node target lesion demonstrated a 38.5% decrease in SUVmax from baseline (Figure 6I). While direct detection of TILT-123 was not assessed in this lesion, the observed reduction in SUVmax is consistent with a potential i.t. effect of TILT-123.

DISCUSSION

This study investigated the safety, efficacy, and biological effects of a fully i.v. split-dose TILT-123 regimen in six patients within the phase 1 clinical trial TUNIMO. While other studies have examined the

are displayed. (C) Volcano plot of significantly upregulated serum proteins detected 1 h post-second dose compared with 1 h post-first dose, across all treatment days. The Mann-Whitney *U* test was used to assess overall differences in protein levels. Proteins with a *p* value <0.05 are displayed. (D) Top five biologically relevant Gene Ontology (GO) terms from enrichment analysis of significantly upregulated serum proteins post-second dose versus post-first dose using ClusterProfiler. Broad terms inherently enriched due to the cytokine-focused panel (e.g., cytokine signaling) and terms not biologically relevant to the therapy were excluded from interpretation. (E) Comparison of fold changes of serum proteins 16 h post-dosing (day 1) between single- and split-dose i.v. patients. The Mann-Whitney *U* test was used to assess overall differences in protein levels. Proteins with a *p* value <0.05 are displayed. Data are presented as mean ± SD. (F) Spearman correlation of post-treatment serum protein levels and OS, grouped by phase of treatment. For proteins showing multiple correlations with OS across different time points, the correlation with the strongest statistical significance was displayed. (G) Top biologically relevant GO terms resulting from GO enrichment analysis of serum proteins positively or negatively associated with OS. Terms present in both groups, with <3 hits, or broadly overlapping (e.g., phosphatidylinositol 3-kinase signaling terms) were filtered. Broad panel-inherent terms (e.g., cytokine signaling) and those not relevant to therapy were excluded.

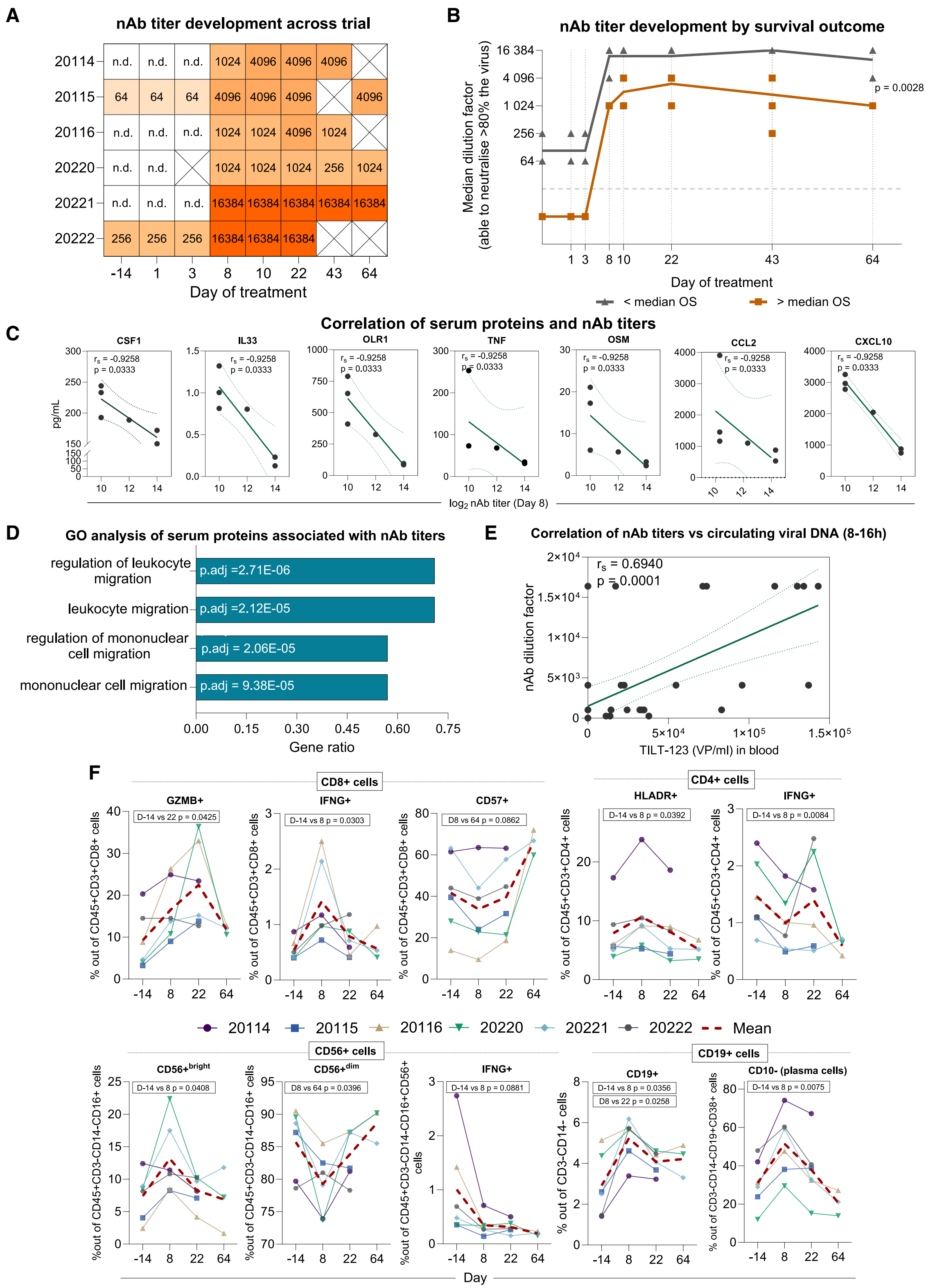

(legend on next page)

safety and therapeutic potential of i.v. administered OVs, to our knowledge, no clinical studies have investigated a split-dosing approach. Additionally, our comparison of single- versus split-i.v. administration of TILT-123 at equivalent doses provides valuable insights into viral bioavailability, which may be extrapolated to other OVs. Our findings show that this approach was feasible, well tolerated, and achieved virus delivery to tumors. Patients showed signs of metabolic disease stabilization by PET criteria, extended OS compared with historical single-dose i.v. + i.t. cohorts, and tumor immune profiles indicative of pro-inflammatory activation. However, given the small and heterogeneous cohort, efficacy observations should be interpreted as exploratory because this study was not powered to demonstrate clinical benefit in a statistical manner.

The most commonly reported AEs were chills, fever, fatigue, and lymphocyte reduction, consistent with the expected immune responses to OV therapy. Similar AE profiles have been reported with i.v. NG-350A, a CD40-armed oncolytic adenovirus administered on days 1, 3, and 5, where fever, chills, and nausea were among the most common treatment-related AEs.[24] In our study, AEs peaked on day 8, possibly due to cumulative dosing and increased systemic exposure. Lymphocyte decreases are frequently observed in viral infections, and a lymphocyte count reduction in blood with a concomitant increase in tumors has been reported to indicate redistribution of lymphocytes to OV-infected sites.[25,26] Transgene-encoded TNF and IL-2 were not elevated in pre-treatment serum days or weeks after the previous injection, suggesting local expression of transgene products without systemic dissemination or long-term persistence in circulation, potentially minimizing off-target effects. Interestingly, we noted a downregulation of proteins related to innate immunity 1 h after the first i.v. dose, which may reflect rapid cytokine and chemokine uptake by responding immune cells rather than reduced production. For example, $CCR2^+$ monocytes internalize CCL2, NK cells *trans*-endocytose IL-15:IL-15 Rα complexes, and decoy receptors such as IL-1R1 sequester IL-1β, collectively contributing to transient decreases in measurable serum proteins.[27–29]

Because adenoviral infections are widespread, preexisting nAbs are expected in a subset of patients but perhaps not frequently toward the chimeric 5/3 capsid, which does not exist in nature. Accordingly, only a minority had detectable nAbs toward the 5/3 capsid at baseline. However, all patients developed nAbs during treatment, with some patients reaching the maximum detectable titers around day 8. Despite this, infectious TILT-123 was detected in blood, suggesting escape from immediate neutralization. One explanation may be that the high levels of circulating virus in blood could not be fully neutralized, keeping in mind that the normal function of nAbs are to protect against small virus amounts shedding into blood from infected epithelia. Patient 20220, lacking baseline nAbs, exhibited high amounts of infectious TILT-123 in the blood. Patient 20115, with high nAbs (1:4,096), showed approximately 8.5-fold lower but still detectable infectious TILT-123 levels (Figure 2G). This aligns with findings from prior clinical studies of i.v. enadenotucirev, a chimeric oncolytic adenovirus, where repeated 21-day dosing cycles (on days 1, 3, and 5) led to reduced detection of infectious virus in blood across cycles, likely due to rising nAb affinity from repeated virus exposure.[30] The sensitivity of our cell-based infectivity assay was limited by factors inherent to blood. Thawed whole blood showed cytotoxicity toward cultured cells, restricting incubation time and potentially reducing viral binding. Of note, Ad5/3 can bind to erythrocytes and lymphocytes, shielding the virus from neutralization while still allowing tumor transduction, potentially resulting in an underestimation of infectious virus titers in the blood.[31]

In our study, higher nAb titers over the course of treatment correlated with lower levels of proteins involved in immune cell migration and shorter OS, suggesting that humoral responses may limit efficacy by promoting viral clearance and dampening immune activation. Interestingly, kinetic analysis of nAb induction revealed that the speed of titer increase may be a relevant variable determining clinical outcomes. Moderate-paced responders achieved increased median OS, whereas both rapid and slow induction of nAbs were observed in patients with worse OS. While the limited sample size restricts definitive interpretation, these observations may suggest that immune response kinetics warrant further investigation. nAb titers also positively correlated with circulating viral DNA, potentially reflecting virus-antibody complex formation or that higher initial viral exposure drives stronger humoral responses.[32] Although immune cell infiltration, viral genomes, and protein were detected in tumors despite nAbs, patients with lower titers showed longer OS, suggesting that higher nAb titers could impair viral delivery in this administration setting. However, the relationship between humoral immune activity and nAb levels in this cohort remains largely correlative. Notably, viral DNA was detected in tumor biopsies from two patients (nos. 20115 and 20220) who exhibited high and low nAb titers, respectively, complicating interpretation of whether nAbs directly limit TILT-123 tumor delivery. Therefore, nAb levels may be more appropriately interpreted as a biomarker of an antiviral-dominant immune response rather than a direct mediator of

Figure 4. Systemic immune dampening by nAbs does not eliminate cellular immune responses

(A) Individual nAb titers of patients across treatment. Titers less than 1:64 were not detectable (n.d.). Time points with unavailable data are marked by X. (B) Comparison of nAb titer development in patients with greater ($n = 3$) versus lower ($n = 3$) than median OS (198 days). Differences between groups were assessed using mixed-effect analysis, where the indicated p value reflects a significant interaction between time points and OS. (C) Spearman correlation of day 8 serum protein levels and nAb titers 16 h post-initial dose. nAb titers were $\log_2$ transformed for data visualization. (D) Top four GO terms associated with serum proteins correlating with nAb titers. (E) Spearman correlation of nAb titers in serum and circulating TILT-123 DNA in whole blood 16 h post-treatment across trial. (F) Flow cytometric analysis of PBMCs collected pre-treatment on baseline and days 8, 22, and 64. Differences between time points were assessed using ordinary one-way ANOVA with Tukey's multiple comparisons test. Results with a p value <0.1 are displayed.

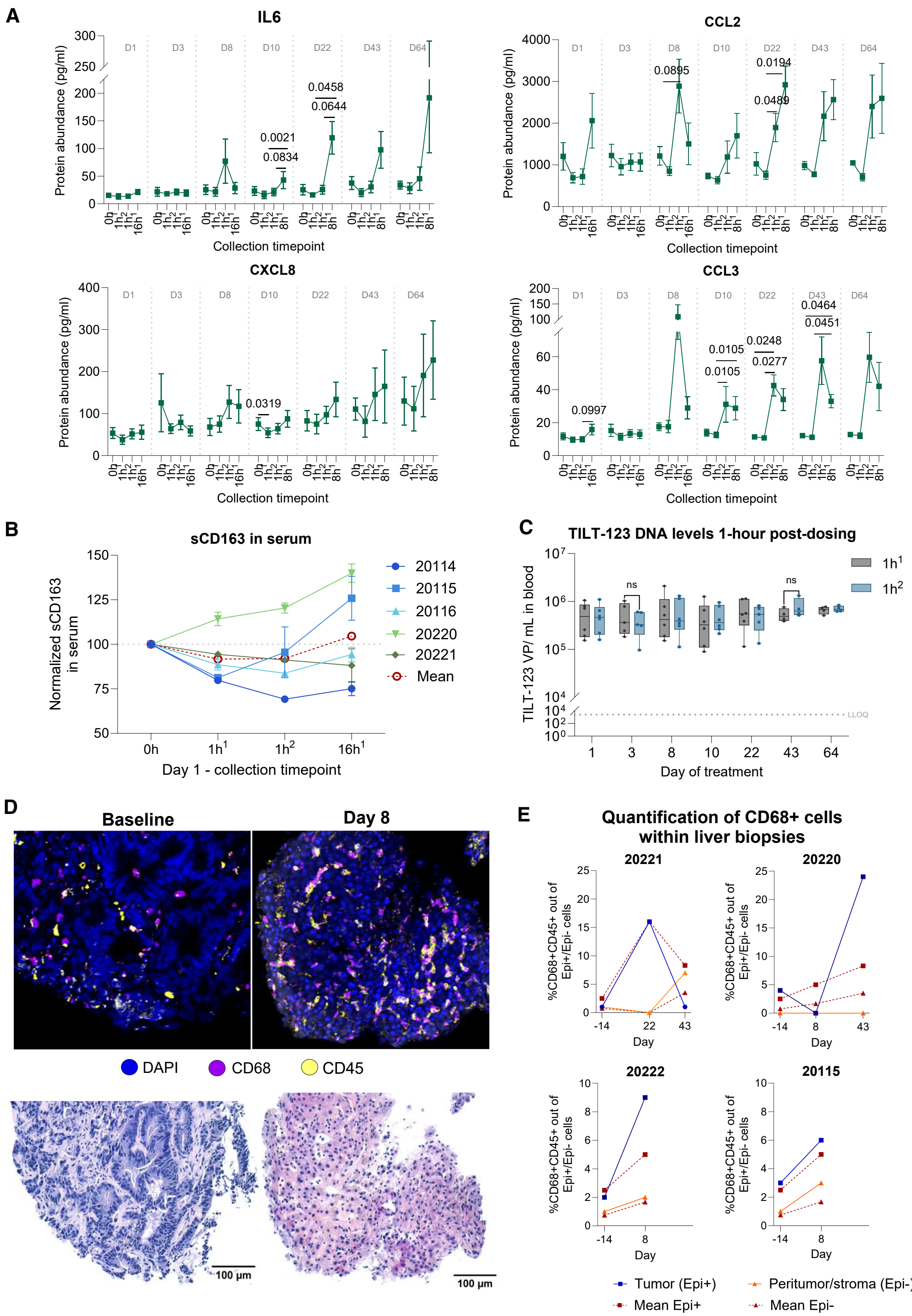

(legend on next page)

reduced delivery to tumors. Interestingly, in the PROTA trial, using TILT-123 i.v. and i.t., both baseline nAb titers and nAb development after treatment were associated with longer OS, highlighting a potential route-dependent effect or a phenomenon specific to ovarian cancer, which may not be apparent in our cohort of heterogeneous tumor types.[33]

While we hypothesized that the second i.v. dose of TILT-123 would result in higher circulating viral DNA levels due to KC depletion by the first dose, qPCR analysis did not support this.[16,17] The possible reasons include insufficient KC saturation, differences between mouse and human macrophage biology, or suboptimal sampling time points. Post-treatment liver tumor biopsies showed modest increases in CD68$^+$ cells within epithelial regions, while quantities of stromal/peritumoral CD68$^+$ cells did not increase to a similar degree, possibly indicating local changes in macrophage-associated markers. While human KC biology is understudied, mouse studies have shown signs of KC zeiosis as early as 15–30 min after i.v. administration of Ad5-based vectors, followed by a >50% loss of KCs at 12 h and recovery by 72 h post-infection.[34] Another study reported that predosing with an adenovirus 4 or 8 h before a second vector enhanced transgene delivery.[35] These reports led us to split the dose approximately 4 h apart in this trial. However, since biopsies were collected several days post-treatment, capturing the relevant early changes in KC may have been suboptimal.

Given that we did not consistently observe higher viral DNA levels after the second administration, additional mechanisms may contribute to the effects of split-dosing. Split-dosing resulted in increased cumulative TILT-123 exposure in AUC analysis, which may enhance both systemic and i.t. biological effects. Additionally, the second TILT-123 dose elicited stronger systemic cytokine responses compared with the first dose; this is consistent with an immune priming-boosting effect, whereby the first dose activates innate immunity and the second dose amplifies inflammatory and antitumor immune responses. This interpretation may be supported by preclinical murine studies with systemic OV administered 48 h apart, which found that the first dose primarily mediates infection, inflammation, and recruitment of innate cells, whereas the second dose modulates the TME and enhances CD8$^+$ T cell immunity despite limited additional infection.[36] Interestingly, we did not observe an association between increased TILT-123 AUC and changes in total SUVmax or OS. This is consistent with previous reports in which the administration of verapamil increased the AUC of adenoviral titers without improving disease control compared with control groups.[37] These findings suggest that the magnitude of systemic viral exposure may not be the primary determinant of disease control by imaging and that the therapeutic benefit of split-dosing might be driven primarily by immune priming rather than by increased viral exposure. Inflammatory swelling of infected tumors may affect imaging-based assessments; however, this would not account for the lack of association with OS. Therefore, further studies in larger patient populations are warranted to interrogate the relationship between AUC and clinical outcomes.

The limited use of T-VEC likely reflects in part the difficulty of i.t. injection in routine clinical practice. In contrast, the i.v. administration of TILT-123 enables targeting of metastatic sites, addressing a critical limitation to the broader clinical adoption of OVs. A key finding was successful tumor delivery of TILT-123 after i.v. administration, confirmed by viral DNA, protein, and immune cell infiltration. Importantly, tumor delivery with split-dose i.v. administration was comparable to the more challenging i.t. administration, suggesting that i.v. dosing does not compromise transduction. However, the evidence for tumor delivery following i.v. TILT-123 remains qualitative and limited. Viral DNA and hexon protein were detected in only a subset of evaluable biopsies, and biopsy numbers were small and collected at variable time points. These limitations should be considered when interpreting viral delivery efficacy and associated immune activation in this cohort. Both TILT-123$^+$ biopsies by qPCR and IHC originated from liver metastases. Since these biopsies were taken 5 days post-treatment, the result likely indicates successful tumor transduction and replication, as non-replicating virus would have been rapidly cleared after administration. Virus detection in tumors likely has low sensitivity, since biopsies only provide a snapshot view, potentially underestimating viral presence if replication and oncolysis occurred outside the sampling window.

Patients with liposarcoma, rectal adenocarcinoma, and PDAC, tumor types with poor prognosis and resistance to standard treatments, appeared to derive the greatest clinical benefit, showing OS above the median and stable metabolic disease or minor metabolic response by PET imaging. The median OS of 198 days observed in this heavily pre-treated cohort compares favorably to the median OS of 109 in the matched single-dose i.v + i.t. cohort.

Assessing OV treatment efficacy with conventional imaging is challenging due to the inflammatory swelling of tumors, known as pseudoprogression.[38] PET imaging may be more sensitive than CT in capturing OV-induced antitumor effects.[39] In this cohort, disease control was observed in 5 out of 6 patients (83.3%) by PET, compared with 2 out of 6 patients (33.3%) by CT, reflecting the

Figure 5. Fluctuations in KC-associated cytokines and limited increase of stromal macrophages may reflect effects of split-dosing
(A) Temporal profiles of markers of KC activation. Data are shown as mean ± SD. Paired comparisons across time points were evaluated using repeated measures one-way ANOVA or Friedman test with Tukey's or Dunn's multiple comparisons test, depending on the data distribution. p values <0.1 are displayed. Days 1–22, n = 5–6, days 43–64, n = 4–6. (B) Normalized sCD163 levels on day 1 of treatment. Data are shown as mean ± SD. Samples were run in technical duplicates. (C) Comparison of TILT-123 DNA levels 1 h post-first and post-second dose. Comparisons between groups were evaluated using two-way ANOVA. Boxes show median and interquartile range; whiskers indicate the minimum and maximum values. (D) mIF staining for CD45$^+$ and CD68$^+$ cells, along with matching H&E staining of a liver tumor biopsy from patient 20221. Black scale bar indicates 100 μm. (E) Quantification of CD45$^+$CD68$^+$ cells within stromal/peritumoral (Epi$^-$) and tumor (Epi$^+$) regions in liver biopsies.

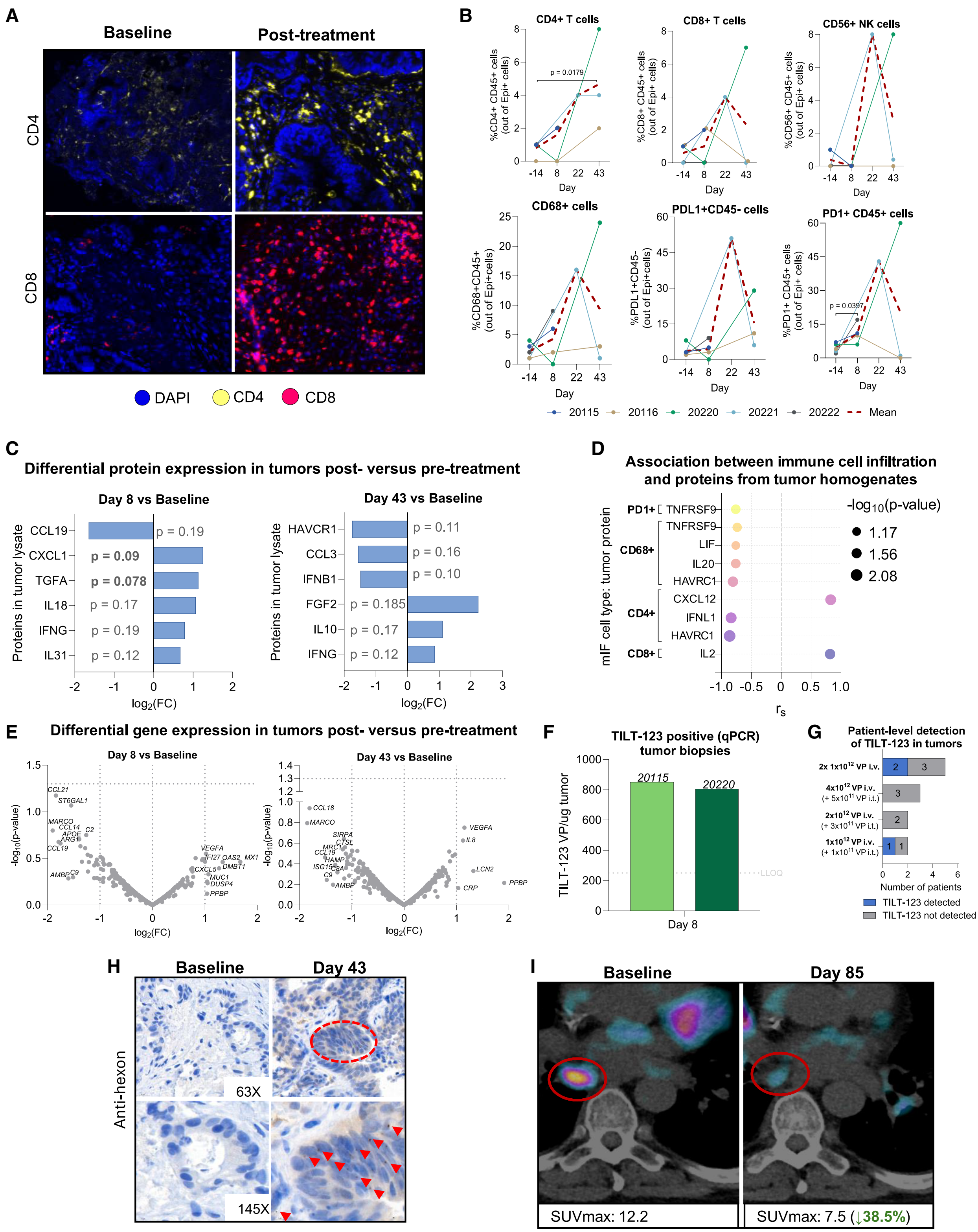

(legend on next page)

limitations of CT imaging in distinguishing true progression from immune-related changes, such as lymphocyte infiltration and inflammation. The higher disease control rate observed by PET likely reflects the ability of metabolic imaging to detect reductions in tumor metabolic activity following OV therapy, whereas inflammatory swelling may obscure treatment responses when assessed using size-based criteria. This disease control rate compares favorably to a phase 1 clinical trial investigating oncolytic herpes simplex virus expressing IL-2/PD-1, where i.v. administration on days 1, 4, 7, 11, 15, and 18 of a 28-day cycle resulted in stable disease (RECIST 1.1) in 3 out of 14 evaluable patients (21.4%).[40] Although patient numbers and treatment regimens differ, our findings suggest encouraging disease stabilization with the split-dose regimen.

Pseudoprogression has been noted in prior TUNIMO cohorts and other immunotherapy trials, including a phase 3 study of T-VEC in melanoma, where over half of the patients initially exhibited lesion enlargement or the appearance of new lesions before responding as assessed by RECIST1.1.[26,41] Although PET imaging can offer greater sensitivity in capturing treatment-induced metabolic changes, it is not without limitations. Fluorodeoxyglucose-PET detects glucose metabolism and therefore cannot distinguish between metabolically active tumor cells and immune cells with high glucose metabolism such as neutrophils, macrophages, and lymphocytes, which are actively recruited to the tumor site following OV administration.[39,42] Consequently, a residual PET signal following OV therapy may partly reflect immune cell activity and TME remodeling rather than tumor burden alone, and in some cases it may resemble immune-related pseudoprogression. This has been demonstrated in a separate clinical trial (TILT-T215) in a metastatic melanoma patient treated with multiple injections of TILT-123 in combination with a single infusion of tumor-infiltrating lymphocytes. Although PET-CT imaging suggested progressive disease, surgical resection and histopathologic analysis revealed no malignant cells, confirming a pathologic complete response.[43]

The study is limited by a small sample size, heterogeneity of tumor types, and differences in sensitivity to immunotherapy, which may affect the generalizability of the findings. Variability in the time of sample collection may also influence observed immune or viral responses. Furthermore, not all biopsies contained sufficient tumor content, impacting the number of samples available for downstream analysis, as was the case for the longest surviving patient. Additional studies with larger, more controlled cohorts would be useful to validate our findings.

In summary, our findings suggest that split-dose i.v. administration of TILT-123 is not only safe and immunologically active but also shows promise as a therapeutic approach, laying the groundwork for future optimization of fully i.v. OV delivery. Successful i.v. delivery would facilitate the development of OVs into a routinely usable approach, addressing a critical need in tumor immunotherapy.

MATERIALS AND METHODS

Patients

Six patients were enrolled into cohort 7 of the TUNIMO trial (ClinicalTrials.gov: NCT04695327) (Table 1). The study was conducted at two sites in Helsinki, Finland: Helsinki University Hospital and Docrates Cancer Center. Key inclusion criteria have been described previously.[26] Informed consent was obtained from all patients prior to trial participation. This study was approved by the Helsinki University Hospital Ethics Committee, approval no. HUS/1804/2020. The clinical trial protocol was reviewed by the Finnish Medical Agency (approval no. 29/2020).

Assessment of treatment response

PET and diagnostic CT imaging of lesions on days 43 and 85 were evaluated by specialized isotope physicians and radiologists, respectively. Lesion response was determined using RECIST 1.1, iRECIST, and PET-based criteria (Table S1). OS and PFS were calculated from enrollment. All patients were deceased by the data cutoff date (January 7, 2025); therefore, no censoring occurred.

Clinical trial protocol

Patients received 1×10^{12} viral particles (VP) of i.v. TILT-123 twice per treatment day on days 1, 3, 8, 10, 22, 43, and 64, with doses given maximally 4 h apart (±2 h). Blood was collected pre-treatment, 1 h after the initial dose ($1h^1$), 1 h after the second dose ($1h^2$), and 8–16 h after the initial dose ($8h^1$–$16h^1$). Urine and saliva were collected pre-treatment and 8–16 h post-treatment. Tumor biopsies were

Figure 6. Fully i.v. administration of TILT-123 results in tumor transduction accompanied by an inflammatory response

(A) mIF imaging of $CD4^+$ and $CD8^+$ T cells in post-treatment tumors. Representative images are shown from patient 20115 (day 8 biopsy, $CD4^+$ staining) and patient 20220 (day 43 biopsy, $CD8^+$ staining). (B) Quantified mIF data of intratumoral immune markers ($CD45^+CD4^+$, $CD45^+CD8^+$, $CD45^+CD56^+$, $CD45^+$, $CD86^+$, $CD45^+PD1^+$, $CD45^-PD\text{-}L1^+$) across visit days. Differences between time points were assessed using the Mann-Whitney *U* test. (C) Expression of proteins from pre- and post-treatment tumor biopsy lysates. Proteins with a *p* value <0.2 (exploratory *p* value) and with log2(FC) >±0.5 are displayed. Statistical differences between groups were assessed using either a *t* test or the Mann-Whitney *U* test. Olink NPX Signature software was used for normalization to yield absolute protein concentrations (pg/mL). Baseline, $n = 5$; day 8, $n = 4$; day 43, $n = 3$. (D) Correlation between immune cell infiltration by mIF and proteins detected within tumor homogenates. Data were generated from days 8, 22, and 43 mIF and tumor proteomics results ($n = 8$ biopsies). (E) Volcano plot of differentially expressed genes analyzed by Nanostring nCounter in pre-treatment versus post-treatment biopsies. Gray dots indicate genes that do not pass threshold values of $p < 0.05$. Baseline, $n = 5$; day 8, $n = 4$; day 43, $n = 3$. (F) Post-treatment liver tumor biopsies positive for TILT-123 DNA, as detected by qPCR. LLOQ for this assay was 250 VP/μg of tissue. (G) Patient summary of overall detection of TILT-123 DNA in injected and noninjected tumor biopsies (by qPCR or hexon staining), stratified by dose. (H) Immunohistochemistry analysis of a metastatic liver lesion from patient 20220. Dashed red circle and red arrows indicate hexon positivity as represented by a speckled brown 3,3′-diaminobenzidine stain. (I) Representative PET images from PDAC patient 20116, showing a fluorodeoxyglucose-avid subcarinal lymph node lesion highlighted by a red circle, before treatment (left, baseline) and on day 85 (right, last imaging time point). Maximum standardized uptake value (SUVmax) is indicated together with the percentage change from baseline.

collected at baseline and pre-treatment on days 8 and 43, if deemed feasible by the investigator. Patient 20221 was biopsied on day 22 instead of day 8. Table S2 summarizes biopsies and corresponding results. The sample collection scheme and trial outline are summarized in Figures 1A and 1B. AEs were reported according to the Common Terminology Criteria for Adverse Events version 5.0.

Patients in this study are primarily compared to patients in the dose-equivalent TUNIMO cohort 5, who received a single i.v. injection of TILT-123 (2×10^{12} VP) on day 1, followed by four rounds of 3×10^{11} VP i.t. injections. Day 1 of treatment represents the most comparable time point as patients received an equivalent i.v. dose.

Viral biodistribution and shedding

DNA was extracted from blood and tumors using the MagMax DNA Multi-Sample Ultra 2.0 Kit (Thermo Fisher, USA) and from urine and saliva using the MagMax Microbiome Ultra Nucleic Acid Isolation Kit (Thermo Fisher). Primer and probe sequences targeting the TILT-123 IRES (internal ribosome entry site)-IL-2 region are listed in Table S3. For bioavailability analysis, cohort 7 results were compared to patients receiving the same i.v. dose in PROTA (ClinicalTrials.gov: NCT05271318) and TUNINTIL (ClinicalTrials.gov: NCT04217473) trials, as only two patients in TUNIMO cohort 5 had available data.

Assessment of infectious particles in blood samples

A549 cells (80,000 cells per well) (American Type Culture Collection [ATCC], USA) were seeded and incubated at 37°C overnight. To limit cell toxicity, serial 10-fold dilutions of blood samples in DMEM (Gibco, USA) were added to monolayers for 1 h at 37°C, followed by washing and overlay with 0.8% of carboxymethyl cellulose (Sigma-Aldrich, USA). After 7 days, cells were fixed, permeabilized, and stained with anti-adenovirus antibody for foci detection.

Serum proteomic analysis

We collected pre-treatment and post-treatment serum and analyzed them using Olink Target 48 Cytokine Panel (Thermo Fisher). Absolute protein expression values were reported in picograms/milliliter.

Flow cytometry analysis of PBMCs

PBMCs were isolated from pre-treatment blood on days 1, 8, 22, and 64. 1×10^6 cells per well were plated, Fc-receptors were -blocked (10 min, 4°C), and cells were stained with antibodies listed in Table S4. Samples were analyzed using Novocyte Quanteon (Agilent Technologies, USA), and data analyzed using FlowJo (version 10.9.0) (BD Biosciences, USA).

Anti-adenovirus antibody analysis

Anti-adenovirus nAbs were measured using a previously established protocol.[44] Complement inactivated patient sera were serially diluted, incubated with Ad5/3-Luc1 for 1 h, and used to infect A549 cells (ATCC) overnight at 37°C. Luciferase expression was measured using a Luciferase Assay System (Promega, USA), and nAb titer was defined as the lowest serum dilution blocking 80% of luciferase expression (range 1:64–1:16,384).

sCD163 ELISA

Serum samples were diluted 1:100, and sCD163 ELISA (Thermo Fisher) was performed according to the manufacturer's instructions.

Multiplex immunofluorescence and IHC staining

Tumor biopsies were formalin fixed and paraffin embedded. Hematoxylin and eosin staining was used to evaluate sample quality and tumor content. Biopsies from patient 20114 were excluded due to the lack of tumor content. A list of primary antibodies used for IHC and mIF can be found in Table S5.

Tumor transcriptomic and proteomic profiling

Tumor samples were homogenized and split for protein and RNA isolation. RNA expression was profiled using Nanostring nCounter (PanCancer Immune Profiling Panel and user-defined Panel Plus). Protein lysates were analyzed with Olink Target 48 Cytokine and Immune Surveillance panels.

Graphical illustrations and statistics

Graphs were produced using GraphPad Prism version 10.1.2 (GraphPad Software, USA), and BioRender. Statistical calculations were performed using GraphPad Prism or RStudio version 4.3.1. The normality of the data was assessed using the Shapiro-Wilk test. Statistical comparisons between groups are specified in the figure legends.

DATA AND CODE AVAILABILITY

Source data are provided with this paper.

ACKNOWLEDGMENTS

The authors thank all the patients participating in the study and their families. The authors thank Minna Oksanen, Susanna Grönberg-Vähä-Koskela, and Sini Raatikainen for their expert assistance. Nanostring and Olink analysis was performed by the FIMM Genomics Targeted Biomarker Assays Unit at the University of Helsinki, supported by HiLIFE and Biocenter Finland. Flow cytometry was performed at the HiLIFE Biomedicum Flow Cytomery Unit at the University of Helsinki. We are grateful for the contributions of the Biomedicum Functional Genomics Unit (FUGU) and the FIMM Digital Microscopy and Molecular Pathology Unit supported by HiLIFE and Biocenter Finland for immunohistochemistry services. This study was supported by the Jane and Aatos Erkko Foundation, TILT Biotherapeutics Oy, the Academy of Finland, HUCH Research Funds (VTR), Cancer Foundation Finland, the Sigrid Juselius Foundation, and the Finnish Red Cross Blood Service. TILT Biotherapeutics was involved in the study design, data analysis, data interpretation, manuscript preparation, and decision to submit the manuscript for publication.

AUTHOR CONTRIBUTIONS

E.J., D.C.A.Q., J.H.A.C., S.A.P., T.V.K., V.A., M.v.d.H., and L.H., investigation, methodology, data acquisition, curation, analysis, and interpretation; S.G.-V.-K., technical and material support; S.S., R.H., C.K., J.M.S., and V.C.-C., experiment design, investigation, methodology, data acquisition, curation, analysis, interpretation, and project supervision; K.J.J., T.A., A.F., J. Kononen, J.S., J. Kemppainen, and M.H., investigation, data acquisition, and interpretation of clinical data; O.H. and A.K. acquired the funding. A.H. was involved in experimental design, investigation, data analysis, interpretation, project supervision, and acquiring the funding. All authors contributed to writing and reviewing the manuscript.

DECLARATION OF INTERESTS

A.H. is a shareholder in Circio Holdings ASA. A.H., V.C.-C., J.H.A.C., and C.K. are employees of and shareholders in TILT Biotherapeutics Oy. O.H. is a shareholder in TILT Biotherapeutics Oy. L.H., D.C.A.Q., R.H., T.V.K., and S.S. are employees of TILT Biotherapeutics. J.M.S. is a TILT Biotherapeutics Oy shareholder, and at the time of the study, he was an employee of TILT Biotherapeutics Oy. K.J.J. has held advisory roles (Bayer AG, Bristol-Myers Squibb, MSD, Roche, Ipsen) and is a shareholder in Faron Pharmaceuticals.

SUPPLEMENTAL INFORMATION

Supplemental information can be found online at https://doi.org/10.1016/j.ymthe.2026.05.007.

REFERENCES

1. Shiravand, Y., Khodadadi, F., Kashani, S.M.A., Hosseini-Fard, S.R., Hosseini, S., Sadeghirad, H., Ladwa, R., O'byrne, K., and Kulasinghe, A. (2022). Immune Checkpoint Inhibitors in Cancer Therapy. Curr. Oncol. *29*, 3044–3060.
2. Motzer, R.J., Escudier, B., McDermott, D.F., George, S., Hammers, H.J., Srinivas, S., Tykodi, S.S., Sosman, J.A., Procopio, G., Plimack, E.R., et al. (2015). Nivolumab versus Everolimus in Advanced Renal-Cell Carcinoma. N. Engl. J. Med. *373*, 1803–1813.
3. Garon, E.B., Hellmann, M.D., Rizvi, N.A., Carcereny, E., Leighl, N.B., Ahn, M.J., Eder, J.P., Balmanoukian, A.S., Aggarwal, C., Horn, L., et al. (2019). Five-Year Overall Survival for Patients With Advanced Non−Small-Cell Lung Cancer Treated With Pembrolizumab: Results From the Phase I KEYNOTE-001 Study. J. Clin. Oncol. *37*, 2518–2527.
4. El-Khoueiry, A.B., Sangro, B., Yau, T., Crocenzi, T.S., Kudo, M., Hsu, C., Kim, T.Y., Choo, S.P., Trojan, J., Welling, T.H., et al. (2017). Nivolumab in patients with advanced hepatocellular carcinoma (CheckMate 040): an open-label, non-comparative, phase 1/2 dose escalation and expansion trial. Lancet *389*, 2492–2502.
5. Russell, S.J., Peng, K.W., and Bell, J.C. (2012). ONCOLYTIC VIROTHERAPY. Nat. Biotechnol. *30*, 658–670.
6. Hemminki, O., Dos Santos, J.M., and Hemminki, A. (2020). Oncolytic viruses for cancer immunotherapy. J. Hematol. Oncol. *13*, 84.
7. Mardi, A., Shirokova, A.V., Mohammed, R.N., Keshavarz, A., Zekiy, A.O., Thangavelu, L., Mohamad, T.A.M., Marofi, F., Shomali, N., Zamani, A., and Akbari, M. (2022). Biological causes of immunogenic cancer cell death (ICD) and anti-tumor therapy; Combination of Oncolytic virus-based immunotherapy and CAR T-cell therapy for ICD induction. Cancer Cell Int. *221*, 168.
8. Macedo, N., Miller, D.M., Haq, R., and Kaufman, H.L. (2020). Clinical landscape of oncolytic virus research in 2020. J. Immunother. Cancer *8*, e001486.
9. Shalhout, S.Z., Miller, D.M., Emerick, K.S., and Kaufman, H.L. (2023). Therapy with oncolytic viruses: progress and challenges. Nat. Rev. Clin. Oncol. *203*, 160–177.
10. Raja, J., Ludwig, J.M., Gettinger, S.N., Schalper, K.A., and Kim, H.S. (2018). Oncolytic virus immunotherapy: future prospects for oncology. J. Immunother. Cancer *6*, 140.
11. Ferguson, M.S., Lemoine, N.R., and Wang, Y. (2012). Systemic Delivery of Oncolytic Viruses: Hopes and Hurdles. Adv. Virol. *2012*, 805629.
12. Shayakhmetov, D.M., Gaggar, A., Ni, S., Li, Z.-Y., and Lieber, A. (2005). Adenovirus Binding to Blood Factors Results in Liver Cell Infection and Hepatotoxicity. J. Virol. *79*, 7478–7491.
13. Worgall, S., Wolff, G., Falck-Pedersen, E., and Crystal, R.G. (1997). Innate immune mechanisms dominate elimination of adenoviral vectors following in vivo administration. Hum. Gene Ther. *8*, 37–44.
14. Hofherr, S.E., Adams, K.E., Chen, C.Y., May, S., Weaver, E.A., and Barry, M.A. (2011). Real-Time Dynamic Imaging of Virus Distribution In Vivo. PLoS One *6*, e17076.
15. Bilzer, M., Roggel, F., and Gerbes, A.L. (2006). Role of Kupffer cells in host defense and liver disease. Liver Int. *26*, 1175–1186.
16. Manickan, E., Smith, J.S., Tian, J., Eggerman, T.L., Lozier, J.N., Muller, J., and Byrnes, A.P. (2006). Rapid Kupffer cell death after intravenous injection of adenovirus vectors. Mol. Ther. *13*, 108–117.
17. Schiedner, G., Hertel, S., Johnston, M., Dries, V., van Rooijen, N., and Kochanek, S. (2003). Selective depletion or blockade of Kupffer cells leads to enhanced and prolonged hepatic transgene expression using high-capacity adenoviral vectors. Mol. Ther. *7*, 35–43.
18. Snoeys, J., Mertens, G., Lievens, J., van Berkel, T., Collen, D., Biessen, E.A.L., and De Geest, B. (2006). Lipid Emulsions Potently Increase Transgene Expression in Hepatocytes after Adenoviral Transfer. Mol. Ther. *13*, 98–107.
19. Tähtinen, S., Kaikkonen, S., Merisalo-Soikkeli, M., Grönberg-Vähä-Koskela, S., Kanerva, A., Parviainen, S., Vähä-Koskela, M., and Hemminki, A. (2015). Favorable alteration of tumor microenvironment by immunomodulatory cytokines for efficient T-Cell Therapy in solid tumors. PLoS One *10*, 1–20.
20. Havunen, R., Siurala, M., Sorsa, S., Grönberg-Vähä-Koskela, S., Behr, M., Tähtinen, S., Santos, J.M., Karell, P., Rusanen, J., Nettelbeck, D.M., et al. (2017). Oncolytic Adenoviruses Armed with Tumor Necrosis Factor Alpha and Interleukin-2 Enable Successful Adoptive Cell Therapy. Mol. Ther. Oncolytics *4*, 77–86.
21. Särkioja, M., Pesonen, S., Raki, M., Hakkarainen, T., Salo, J., Ahonen, M.T., Kanerva, A., and Hemminki, A. (2008). Changing the adenovirus fiber for retaining gene delivery efficacy in the presence of neutralizing antibodies. Gene Ther. *15*, 921–929.
22. Bieghs, V., and Trautwein, C. (2013). The innate immune response during liver inflammation and metabolic disease. Trends Immunol. *34*, 446–452.
23. Ragab, H.M., El Maksoud, N.A., Amin, M.A., and Elaziz, W.A. (2021). Performance of serum CD163 as a marker of fibrosis in patients with NAFLD. Diabetes Metab. Syndr. *15*, 87–92.
24. Naing, A., Khalil, D., Rosen, O., Camidge, D.R., Lillie, T., Ji, R.R., Stacey, A., Thomas, M., and Rosen, L. (2024). First-in-human clinical outcomes with NG-350A, an anti-CD40 expressing tumor-selective vector designed to remodel immunosuppressive tumor microenvironments. J. Immunother. Cancer *12*, e010016.
25. Jafarzadeh, A., Jafarzadeh, S., Nozari, P., Mokhtari, P., and Nemati, M. (2021). Lymphopenia an important immunological abnormality in patients with COVID-19: Possible mechanisms. Scand. J. Immunol. *93*, e12967.
26. Pakola, S.A., Peltola, K.J., Clubb, J.H.A., Jirovec, E., Haybout, L., Kudling, T.V., Alanko, T., Korpisaari, R., Juteau, S., Jaakkola, M., et al. (2024). Safety, Efficacy, and Biological Data of T-Cell-Enabling Oncolytic Adenovirus TILT-123 in Advanced Solid Cancers from the TUNIMO Monotherapy Phase I Trial. Clin Cancer Res *30*, 3715–3725. https://doi.org/10.1158/1078-0432.CCR-23-3874.
27. Zhao, B.N., Campbell, J.J., Salanga, C.L., Ertl, L.S., Wang, Y., Yau, S., Dang, T., Zeng, Y., McMahon, J.P., Krasinski, A., et al. (2019). CCR2-Mediated Uptake of Constitutively Produced CCL2: A Mechanism for Regulating Chemokine Levels in the Blood. J. Immunol. *203*, 3157–3165.
28. Bourke, E., Cassetti, A., Villa, A., Fadlon, E., Colotta, F., and Mantovani, A. (2003). IL-1β Scavenging by the Type II IL-1 Decoy Receptor in Human Neutrophils. J. Immunol. *170*, 5999–6005.
29. Anton, O.M., Peterson, M.E., Hollander, M.J., Dorward, D.W., Arora, G., Traba, J., Rajagopalan, S., Snapp, E.L., Garcia, K.C., Waldmann, T.A., and Long, E.O. (2020). Trans-endocytosis of intact IL-15Rα–IL-15 complex from presenting cells into NK cells favors signaling for proliferation. Proc. Natl. Acad. Sci. *117*, 522–531.
30. Machiels, J.P., Salazar, R., Rottey, S., Duran, I., Dirix, L., Geboes, K., Wilkinson-Blanc, C., Pover, G., Alvis, S., Champion, B., et al. (2019). A phase 1 dose escalation study of the oncolytic adenovirus enadenotucirev, administered intravenously to patients with epithelial solid tumors (EVOLVE). J. Immunother. Cancer *7*, 20.
31. Zafar, S., Quixabeira, D.C.A., Kudling, T.V., Cervera-Carrascon, V., Santos, J.M., Grönberg-Vähä-Koskela, S., Zhao, F., Aronen, P., Heiniö, C., Havunen, R., et al. (2020). Ad5/3 is able to avoid neutralization by binding to erythrocytes and lymphocytes. Cancer Gene Ther. *285*, 442–454.
32. Allen, R.J., and Byrnes, A.P. (2019). Interaction of adenovirus with antibodies, complement, and coagulation factors. FEBS Lett. *593*, 3449–3460.
33. Block, M.S., Clubb, J.H.A., Mäenpää, J., Pakola, S., Quixabeira, D.C.A., Kudling, T., Jirovec, E., Haybout, L., van der Heijden, M., Zahraoui, S., et al. (2025). The oncolytic adenovirus TILT-123 with pembrolizumab in platinum resistant or refractory ovarian cancer: the phase 1a PROTA trial. Nat. Commun. *16*, 1–14.
34. Naumenko, V.A., Vishnevskiy, D.A., Stepanenko, A.A., Sosnovtseva, A.O., Chernysheva, A.A., Abakumova, T.O., Valikhov, M.P., Lipatova, A.V., Abakumov,

M.A., and Chekhonin, V.P. (2022). In Vivo Tracking for Oncolytic Adenovirus Interactions with Liver Cells. Biomedicines *10*, 1697.

35. Tao, N., Gao, G.P., Parr, M., Johnston, J., Baradet, T., Wilson, J.M., Barsoum, J., and Fawell, S.E. (2001). Sequestration of Adenoviral Vector by Kupffer Cells Leads to a Nonlinear Dose Response of Transduction in Liver. Mol. Ther. *3*, 28–35.

36. Naumenko, V., Rajwani, J., Turk, M., Zhang, C., Tse, M., Davis, R.P., Kim, D., Rakic, A., Dastidar, H., Van, S., et al. (2022). Repeated dosing improves oncolytic rhabdovirus therapy in mice via interactions with intravascular monocytes. Commun. Biol. *5*, 1385.

37. Koski, A., Raki, M., Nokisalmi, P., Liikanen, I., Kangasniemi, L., Joensuu, T., Kanerva, A., Pesonen, S., Alemany, R., and Hemminki, A. (2012). Verapamil Results in Increased Blood Levels of Oncolytic Adenovirus in Treatment of Patients With Advanced Cancer. Mol. Ther. *20*, 221–229.

38. Andtbacka, R.H.I., Ross, M., Puzanov, I., Milhem, M., Collichio, F., Delman, K.A., Amatruda, T., Zager, J.S., Cranmer, L., Hsueh, E., et al. (2016). Patterns of Clinical Response with Talimogene Laherparepvec (T-VEC) in Patients with Melanoma Treated in the OPTiM Phase III Clinical Trial. Ann. Surg. Oncol. *23*, 4169–4177.

39. Koski, A., Ahtinen, H., Liljenback, H., Roivainen, A., Koskela, A., Oksanen, M., Partanen, K., Laasonen, L., Kairemo, K., Joensuu, T., and Hemminki, A. (2013). [18F]-Fluorodeoxyglucose Positron Emission Tomography and Computed Tomography in Response Evaluation of Oncolytic Adenovirus Treatments of Patients with Advanced Cancer. Hum. Gene Ther. *24*, 1029–1041. https://home.liebertpub.com/hum.

40. Niu, J., Barve, M.A., Spira, A.I., Edenfield, W.J., Wang, K., Liu, G., McKean, M., Fu, W., Qiu, M., and Cheng, P. (2024). Intravenous infusion (IV) or intracavitary perfusion (IP) of T3011, an oncolytic HSV expressing IL-12 and PD-1 antibody: Analyses of two phase 1 studies. J. Clin. Oncol. *42*, e14581.

41. Andtbacka, R.H.I., Kaufman, H.L., Collichio, F., Amatruda, T., Senzer, N., Chesney, J., Delman, K.A., Spitler, L.E., Puzanov, I., Agarwala, S.S., et al. (2015). Talimogene laherparepvec improves durable response rate in patients with advanced melanoma. J. Clin. Oncol. *33*, 2780–2788.

42. Rahman, W.T., Wale, D.J., Viglianti, B.L., Townsend, D.M., Manganaro, M.S., Gross, M.D., Wong, K.K., and Rubello, D. (2019). The impact of infection and inflammation in oncologic 18F-FDG PET/CT imaging. Biomed. Pharmacother. *117*, 109168.

43. Monberg, T.J., Kudling, T., Albieri, B., Pakola, S., Ellebaek, E., Donia, M., Eefsen, R.L., von Buchwald, C., Kistler, C., Santos, J.M., et al. (2024). Durable complete response after combined treatment with tumor-infiltrating lymphocytes and oncolytic adenovirus (TILT-123) in a patient with metastatic mucosal melanoma. Immunooncol. Technol. *24*, 100726.

44. Hemminki, A., Wang, M., Desmond, R.A., Strong, T.V., Alvarez, R.D., and Curiel, D.T. (2002). Serum and Ascites Neutralizing Antibodies in Ovarian Cancer Patients Treated with Intraperitoneal Adenoviral Gene Therapy. Hum. Gene Ther. *13*, 1505–1514. https://home.liebertpub.com/hum.

YMTHE, Volume *34*

Supplemental Information

Fully intravenous split-dose administration of the oncolytic adenovirus TILT-123 in advanced solid tumors

Elise Jirovec, Katriina J. Jalkanen, Dafne C.A. Quixabeira, James H.A. Clubb, Tuomo Alanko, Tatiana V. Kudling, Victor Arias, Santeri A. Pakola, Susanna Grönberg-Vähä-Koskela, Mirte van der Heijden, Anniina Färkkilä, Juha Kononen, Jorma Sormunen, Jukka Kemppainen, Maria Hellesuo, Lyna Haybout, Claudia Kistler, Suvi Sorsa, Riikka Havunen, Joao M. Santos, Anna Kanerva, Victor Cervera-Carrascon, Otto Hemminki, and Akseli Hemminki

Table S1. PET-based criteria used for evaluation of antitumor efficacy.

Complete metabolic response (CMR)	Complete resolution of fluorodeoxyglucose (FDG) activity within measurable lesions and all reliably assessable lesions to background levels. No new FGD-avid lesions in pattern typical of cancer*. Lymph nodes may remain metabolically active due to immune response (activated lymphocytes take up FDG)
Partial metabolic response (PMR)	>30% reduction in FDG activity measured as the summed SUVmax of measurable lesions (up to five lesions, max 2/organ). No new FGD avid lesions in pattern typical of cancer*.
Minor metabolic response (MMR)	10-29% decrease in FGD summed SUVmax. No new FDG-avid lesions in pattern typical of cancer*.
Stable metabolic disease (SMD)	0-9% decrease or up to <30% increase in FDG summed SUVmax. No new FDG-avid lesions in patten typical of cancer*.
Progressive metabolic disease (PMD)	≥30% increase in FDG summed SUVmax in pattern typical of tumor, or new clearly FDG-avid clinically significant lesions in pattern typical of cancer*.

* Defined as lesions associated with CT abnormality most consistent with cancer ≥2 cm in diameter, and clearly not because of inflammation related to treatment response.

Table S2. Summary of collected and analyzed tumor biopsies. Each column reflects individual biopsy results per day of collection for a specific assay. The final column summarizes overall positivity, defined as a positive result in at least one of the three assays. + indicates a collected biopsy or positive result, - indicates not collected or negative result.

Patient ID	Tumor biopsy location	Tumor biopsy collected D-14/D8/D43	TILT-123 DNA detection by qPCR	Hexon detection by IHC	Immune cell infiltration by mIF	Positivity in any assay
20114	Thorax	+/+/-	Excluded - no tumor present			
20115	Liver	+/+/-	D8+	D8-	D8+	D8+
20116	Lung	+/+/+	D8- D43 -	D8- D43-	D8+ D43+	D8+D43+
20220	Liver	+/+/+	D8+ D43-	D8+ D43+	D8- D43+	D8+D43+
20221	Liver	+/*/+	D22- D43-	D22- D43-	D22+ D43-	D22+D43-
20222	Liver	+/+/-	D8-	D8-	D8+	D8+

*Biopsy taken on D22 instead of D8

Table S3. Primer and probe sequences used for qPCR detection of TILT-123 DNA.

	Target Sequence
Forward primer	5'- CATGCTTTACA TGTGTTTAGTC GAG-3'
Reverse primer	5'- TAGTGCAATGC AAGACAGGAG-3'
Probe	5'-/56- FAM/TTGCATCC T/ZEN/GTACATG GTTGTGGC/3IABkFQ/ -3'

Table S4. Antibodies used in flow cytomery analysis of PBMCs.

Antibody	Clone	Conjugation	Supplier
CD45	2D1	APC-H7	BD
CD3	OKT3	FITC<br>BV711	BioLegend
CD8	SK1	BV510	BD
CD4	OKT4	BV605	BioLegend
Gzmb	QA16A02	APC	BioLegend
CD57	NK-1	PE-CF594	BD
IFNG	4S.B3	FITC	BioLegend
HLADR	L243	BV570	BioLegend
CD56	B159	PE-Cy5	BD
CD57	NK-1	PE-CF594	BD
CXCR3	G025H7	PE-Cy7	BioLegend
CXCR5	L138D7	BV650	BioLegend
CD16	3G8	BV570	BioLegend
CD14	HCD14	FITC	BioLegend
CD19	HIB19	BV650	BioLegend
CD10	HI10a	PE-CF594	BD

Table S5. List of primary antibodies used in IHC and mIF studies.

Target	Dilution	Catalog number	Supplier
CD68	1:1000	ab314474	Abcam
CD56	1:100	156R-94	Cell Marque
CD8	1:300	M7103	Dako
CD4	1:400	ab133616	Abcam
PD-1	1:150	LSB12784	LSBio
CD45	1:100	M0701	Dako
CD20	1:200	MS-340	ThermoFisher
PD-L1	1:200	13684	Cell Signaling Techonology
Pan cytokeratin	1:200	ab9377	Abcam
Hexon	1:1000	ab1056	Millipore

Table S6. Adverse events reported according to the Common Terminology Criteria for Adverse Events (CTCAE) v5.0. Clinician-reported events were mapped to the corresponding CTCAE terms, exceptions were kept as reported when no suitable CTCAE term existed (e.g. subfebrile body temperature).

TILT-123 related adverse event	Number of treatment-related adverse events, n (% of all AEs)	Grade 1	Grade 2	Grade 3	Number of unique patients
Chills	14 (31.8)	11 (25)	3 (6.8)	0	6/6 (100)
Fever	10 (22.7)	5 (11.4)	4 (9.1)	1 (2.3)	4/6 (66.7)
Fatigue	4 (9.1)	3 (6.8)	1 (2.3)	0	3/6 (50)
Flu-like symptoms	1 (2.3)	1 (2.3)	0	0	1/6 (16.7)
Subfebrile body temperature	1 (2.3)	1 (2.3)	0	0	1/6 (16.7)
Diarrhea	1 (2.3)	1 (2.3)	0	0	1/6 (16.7)
Headache	1 (2.3)	1 (2.3)	0	0	1/6 (16.7)
Rhinitis	1 (2.3)	1 (2.3)	0	0	1/6 (16.7)
Syncope	1 (2.3)	0	0	1 (2.3)	1/6 (16.7)
Alanine aminotransferase increased	2 (4.5)	2 (4.5)	0	0	2/6 (33.3)
Alkaline phosphatase increased	1 (2.3)	1 (2.3)	0	0	1/6 (16.7)
Aspartate aminotransferase increased	2 (4.5)	2 (4.5)	0	0	2/6 (33.3)
Blood bilirubin increased	1 (2.3)	1 (2.3)	0	0	1/6 (16.7)
Lymphocyte count decreased	4 (9.1)	0	2 (4.5)	2 (4.5)	2/6 (33.3)
Total	**44**	**30**	**10**	**4**	

Table S7. Adverse events reported according to the CTCAE v5.0. Clinician-reported events were mapped to the corresponding CTCAE terms, exceptions were kept as reported when no suitable CTCAE term existed (e.g. subfebrile body temperature, pain at biopsy site) or exact subtype was not specified (e.g. tachycardia). Related events (e.g. shingles, shingle pain, post-herpetic pain) were grouped under the single CTCAE term 'Shingles/herpes zoster' for consistency.

Adverse Event	Grade 1	Grade 2	Grade 3	Grade 4	Grade 5	Total
Chills	11	3	0	0	0	14
Cough	3	0	0	0	0	3
Fever	5	4	1	0	0	10
Rhinitis	2	0	0	0	0	2
Subfebrile body temperature	1	0	0	0	0	1
Fatigue	3	2	0	0	0	5
Flu-like symptoms	1	0	0	0	0	1
Syncope	0	0	1	0	0	1
Headache	2	0	0	0	0	2
Sinusitis	0	1	0	0	0	1
Nausea	2	0	0	0	0	2
Anorexia	2	0	0	0	0	2
Ear pain	2	0	0	0	0	2
Constipation	0	1	0	0	0	1
Diarrhea	1	0	0	0	0	1
Dyspepsia	0	1	0	0	0	1
Dysphagia	1	0	0	0	0	1
Hypertension	0	1	0	0	0	1
Tachycardia	1	0	0	0	0	1
Superficial thromboflebitis	1	0	0	0	0	1
ALT increased	2	0	0	0	0	2
ALP increased	1	0	0	0	0	1
AST increased	2	1	0	0	0	3
Blood bilirubin increased	1	0	0	0	0	1
Lymphocyte count decreased	0	2	2	0	0	4
Myalgia	2	0	0	0	0	2
Abdominal pain	1	2	1	0	0	4
Pain in biopsy site	1	1	0	0	0	2
Fall	1	0	0	0	0	1
Eczema	1	0	0	0	0	1
Pleural effusion	0	1	0	0	0	1
Shingles/herpes zoster	4	2	0	0	0	6

Table S8

Source data for all figures and tables are provided in a separate Excel file titled 'Table S8'

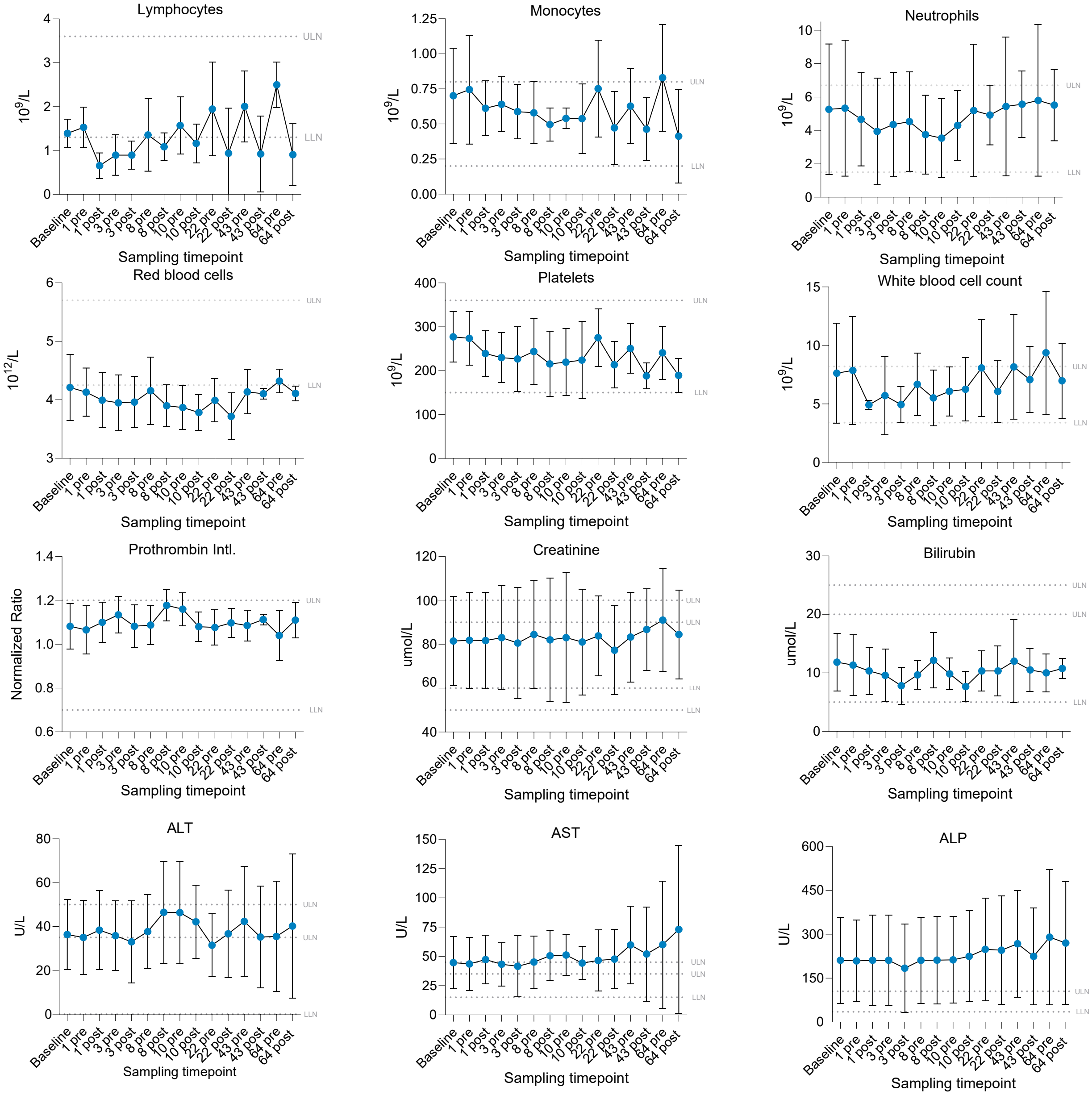

Figure S1. Summary of clinical hematological values throughout the trial. Data are presented as mean ± SD. ULN = upper limit of normal, LLN = lower limit of normal. Baseline to day 43 pre-dosing n = 5-6 patients, day 43 post-dosing to day 64 n = 4 patients.

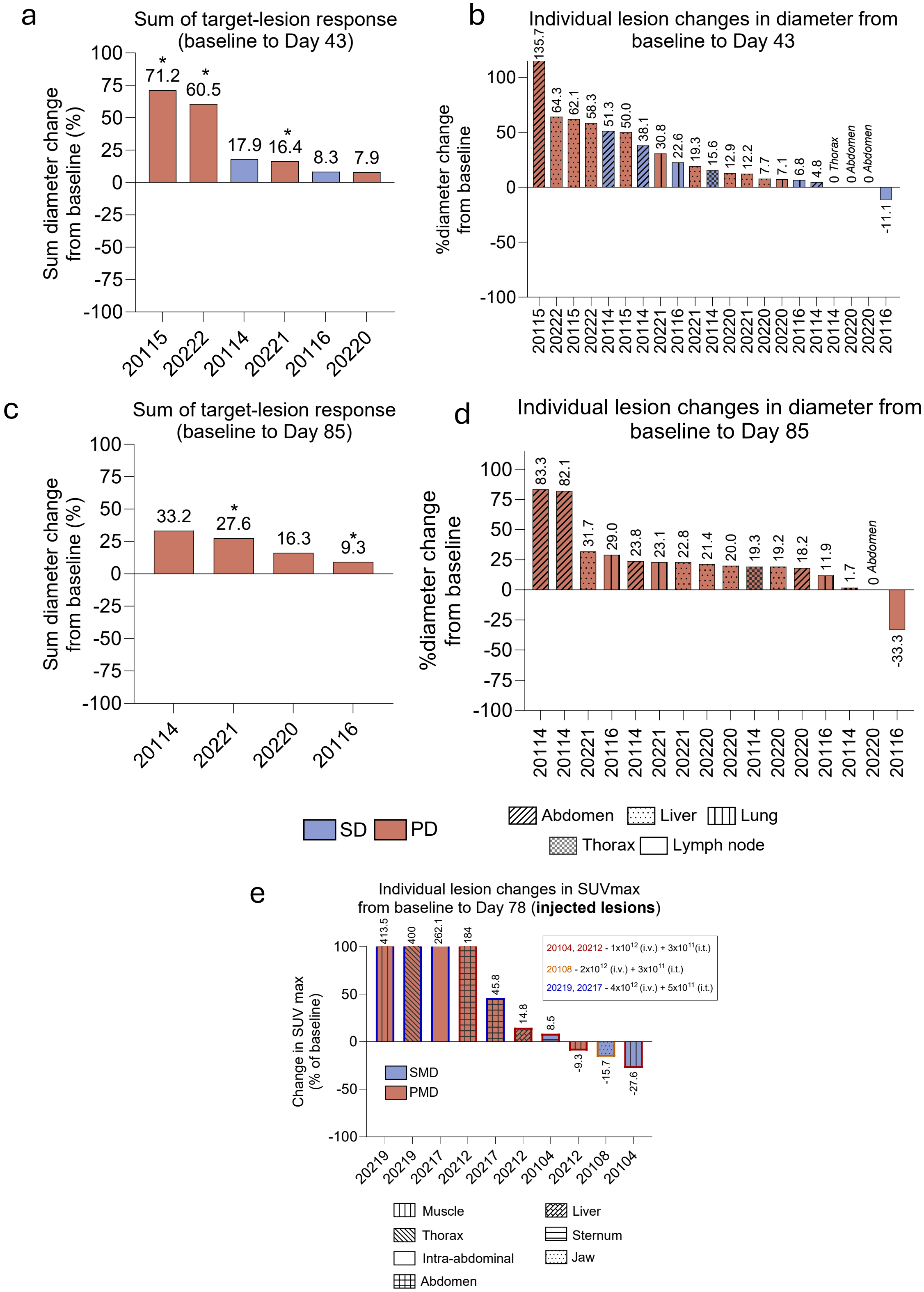

Figure S2. CT and PET-based evluation of target lesions responses across cohorts. a-d) Evaluation of target lesions using CT imaging. * Indicates development of new lesions. SD= stable disease, PD = progressive metabolic disease. e) Individual lesions changes evaluated by PET imaging from patients treated in historical i.t. + i.v. cohorts. SMD = stable metabolic disease, PMD = progressive metabolic disease.

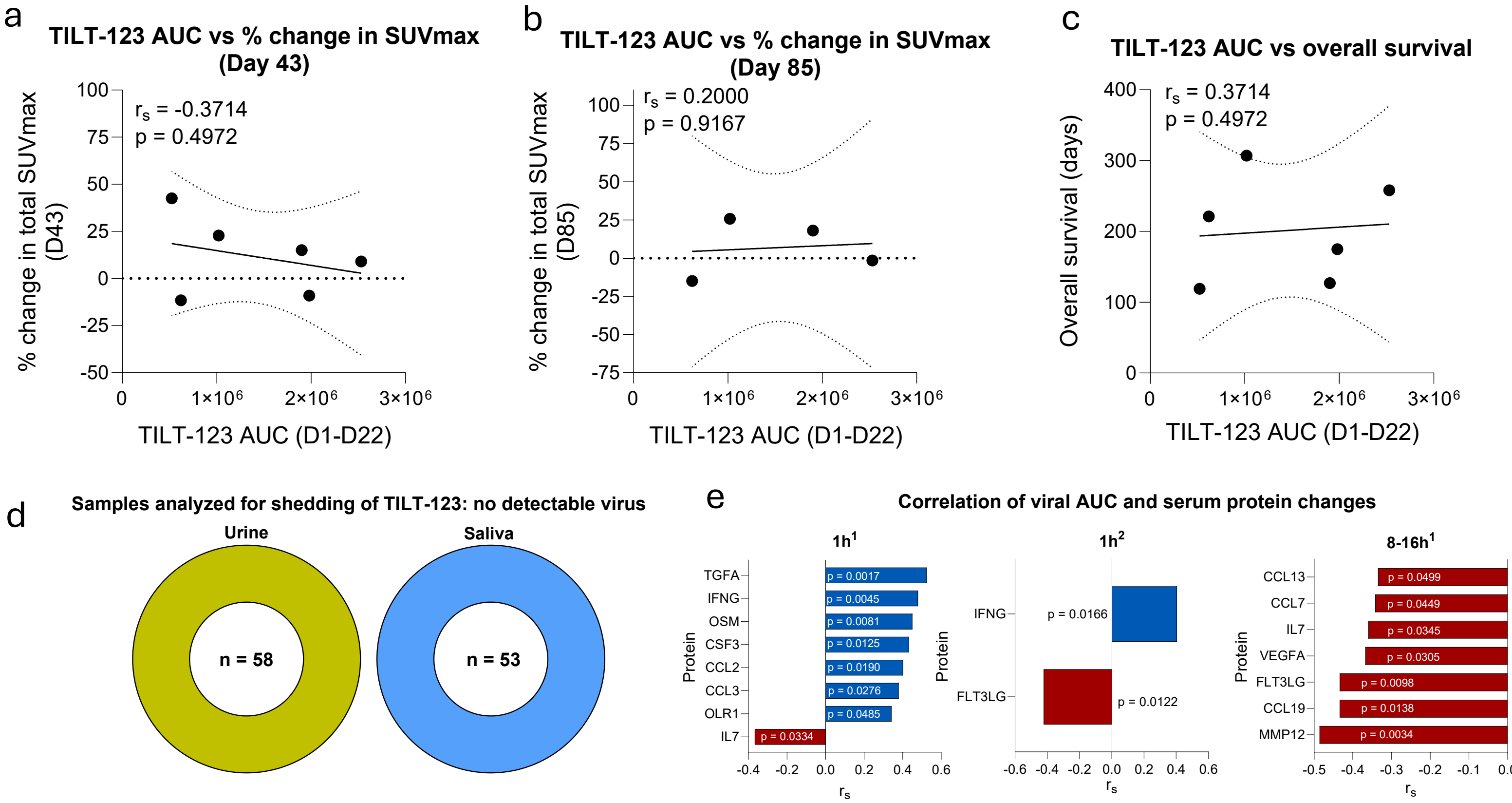

Figure S3. TILT-123 exposure in relation to clinical and proteomic changes, with a summary of shedding data. a-b) Spearman correlation between percentage change in total SUVmax at Day 43 (a) and Day 85 (b) and TILT-123 AUC from Days 1-22. c) Spearman correlation between OS and TILT-123 AUC from Days 1-22. d) Summary of shedding samples analyzed for presence of TILT-123. No virus levels surpassing the LLOQ of 4380 VP/mL were detected. e) Spearman correlation between TILT-123 AUC and serum protein changes post-treatment. Viral DNA AUC was calculated for each treatment day, and correlations were performed seperately for each protein and timepoint.

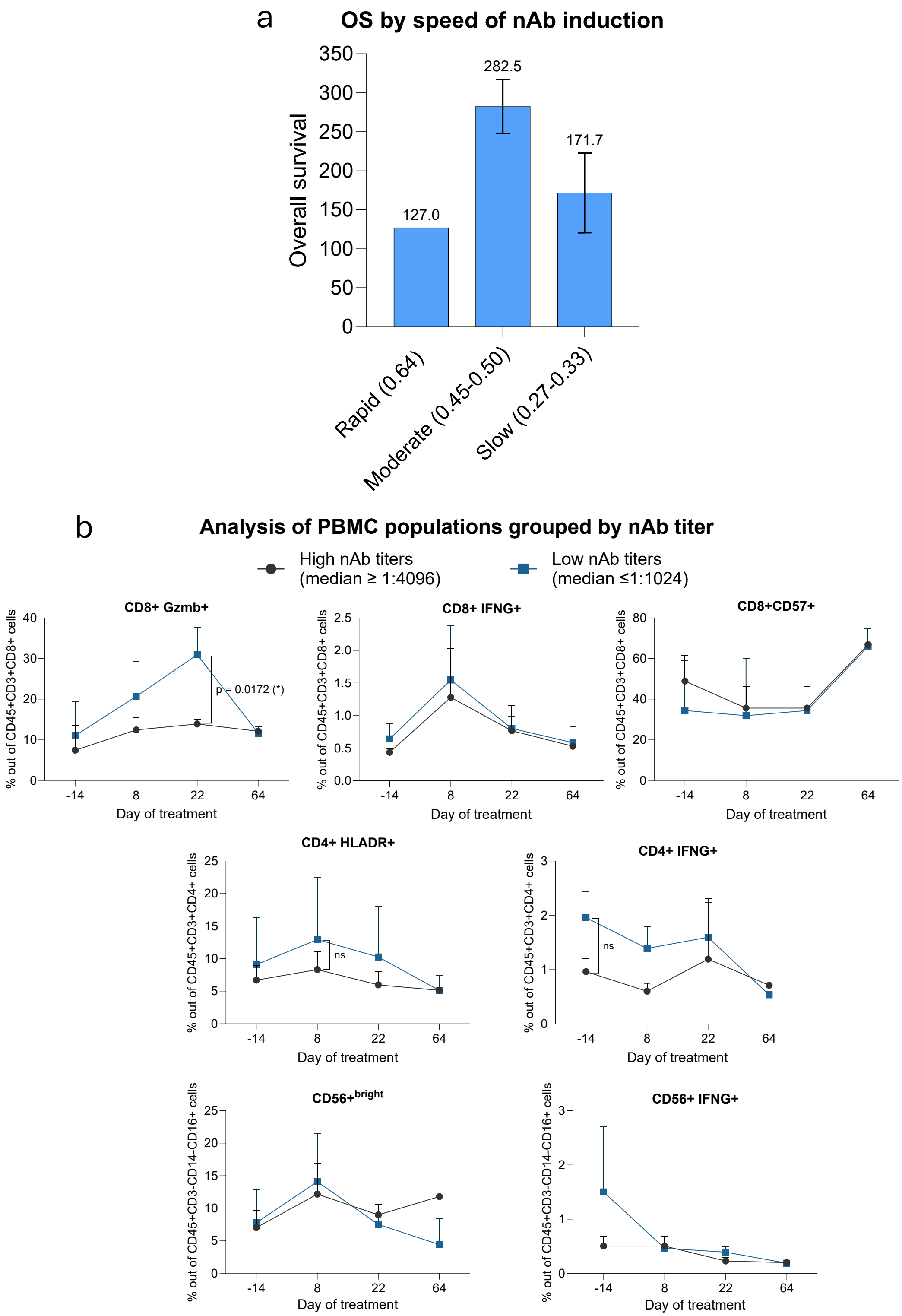

Figure S4. Characterization of nAb kinetics and immune parameters. a) Patient overall survival grouped by speed of nAb induction. Speed of nAb induction was calculated as the log2-fold change from baseline to peak titer divided by the number of days to reach the peak. b) Analysis of PBMC populations grouped by median nAb titer achieved while on treatment. Data are shown as mean ± SD.

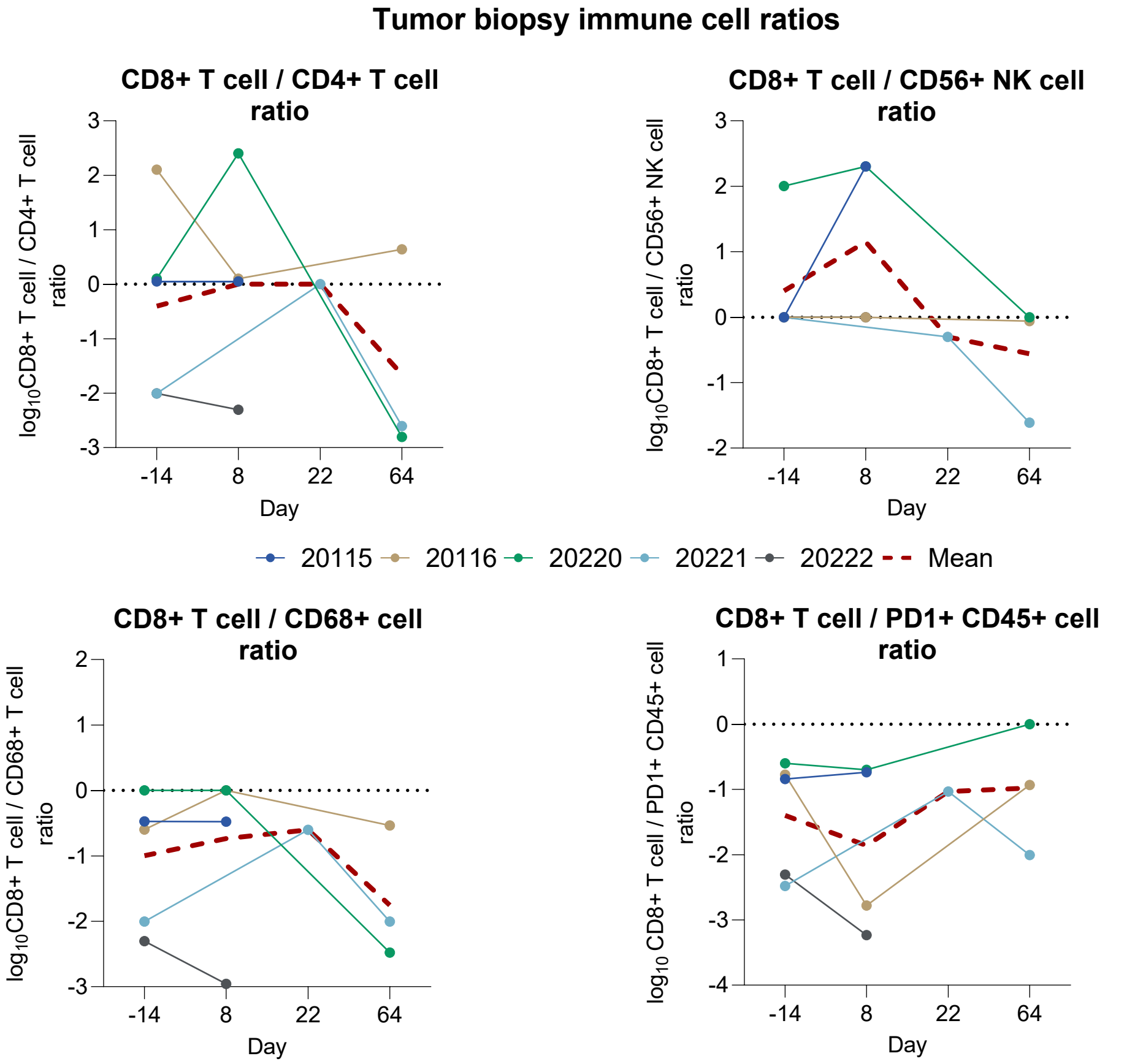

Figure S5. Immune cell ratio analysis in patient tumor biopsies. Individual lines indicate patient biopsies taken pre-treatment (Day -14) and post-treatment (Days 8, 22, and 64). Log_{10} ratios of immune cell populations are shown, where positive values indicate the numerator is more abundant than the denominator, negative values indicate the numerator is less abundant, and zero indicates an equal proportion of both populations. A pseudocount (0.01) was added to all values before to avoid division by zero.
